# Correction: A probabilistic model for the ultradian timing of REM sleep in mice

**DOI:** 10.1371/journal.pcbi.1010225

**Published:** 2022-06-02

**Authors:** Sung-Ho Park, Justin Baik, Jiso Hong, Hanna Antila, Benjamin Kurland, Shinjae Chung, Franz Weber

The links provided in the Data Availability statement for this paper are no longer correct. The correct statement is: All data files are available for download at the following URLs: https://zenodo.org/record/5817119#.YdvvNC_kGTc and https://zenodo.org/record/5820559#.Ydvvcy_kGTc.

## References

[1] Park SH, Baik J, Hong J, Antila H, Kurland B, et al. (2021) A probabilistic model for the ultradian timing of REM sleep in mice. PLOS Computational Biology 17(8): e1009316. 10.1371/journal.pcbi.1009316 34432801 PMC8423363

