# Supplementary figures and images for: A probabilistic model for the ultradian timing of REM sleep in mice
Source: PLoS Comput Biol. 2021 Aug 25;17(8):e1009316. doi: 10.1371/journal.pcbi.1009316 (PMC8423363; doi:10.1371/journal.pcbi.1009316)

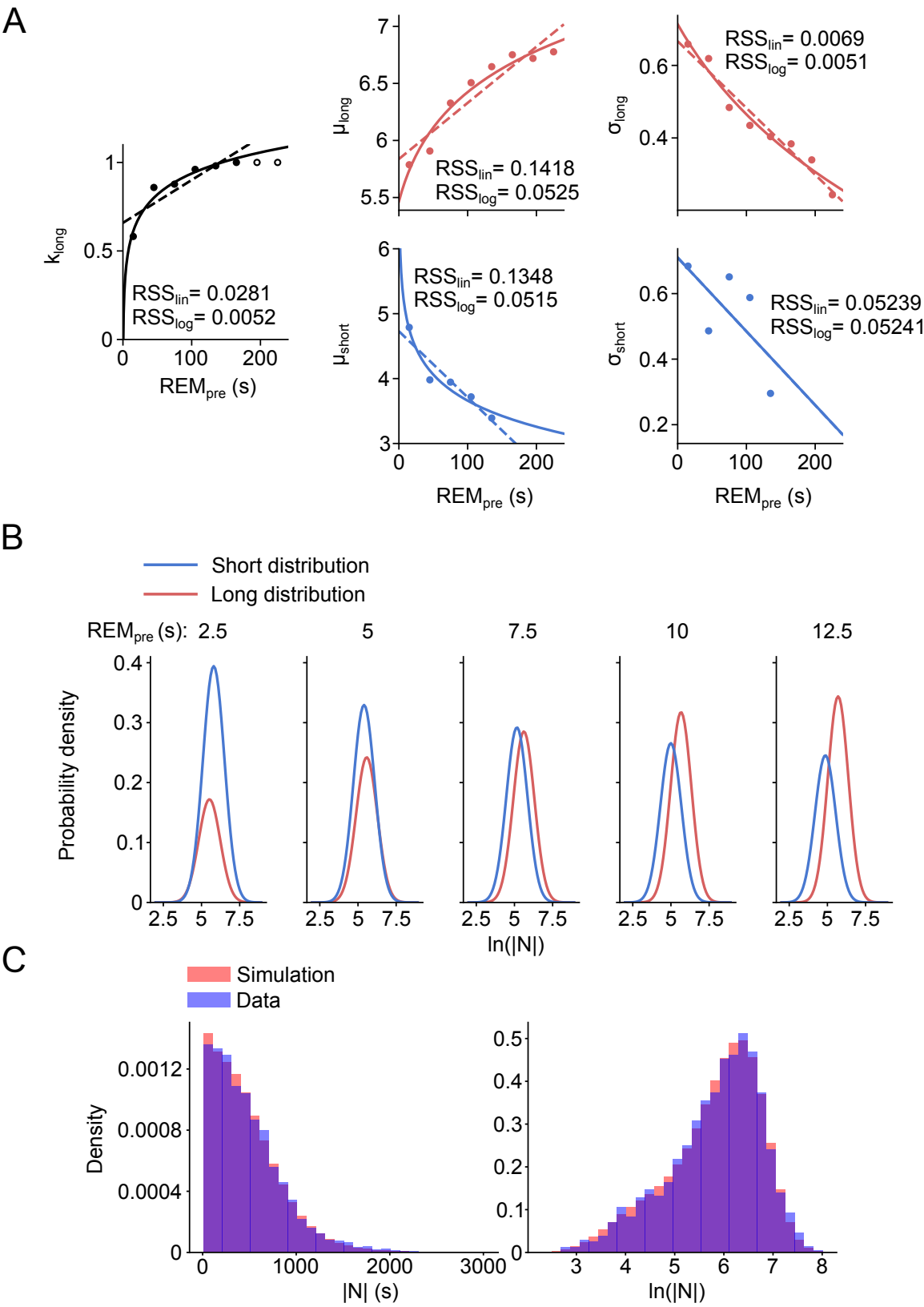

S1 Fig. Conditional GMM for the light phase and model validation.

Supplement: S1 Fig — (A) Comparison of logarithmic (solid lines) and linear (dashed lines) fits for the functions describing the relationship between REMpre and each GMM parameter. For klong, we only fitted the functions to the first 6 REMpre bins (filled circles). For the remaining REMpre bins, klong was set to 1. (B) Estimated probability density functions (PDFs) of the Gaussian distributions for short and long cycles for each 2.5 s increment of REMpre in the range 2.5 s—12.5 s. (C) Histogram over |N| on the normal (left) and natural log scale (right). The histogram for the actual data is compared with the prediction by the GMM (10,000 model simulations). (PDF) [file pcbi.1009316.s001.pdf]

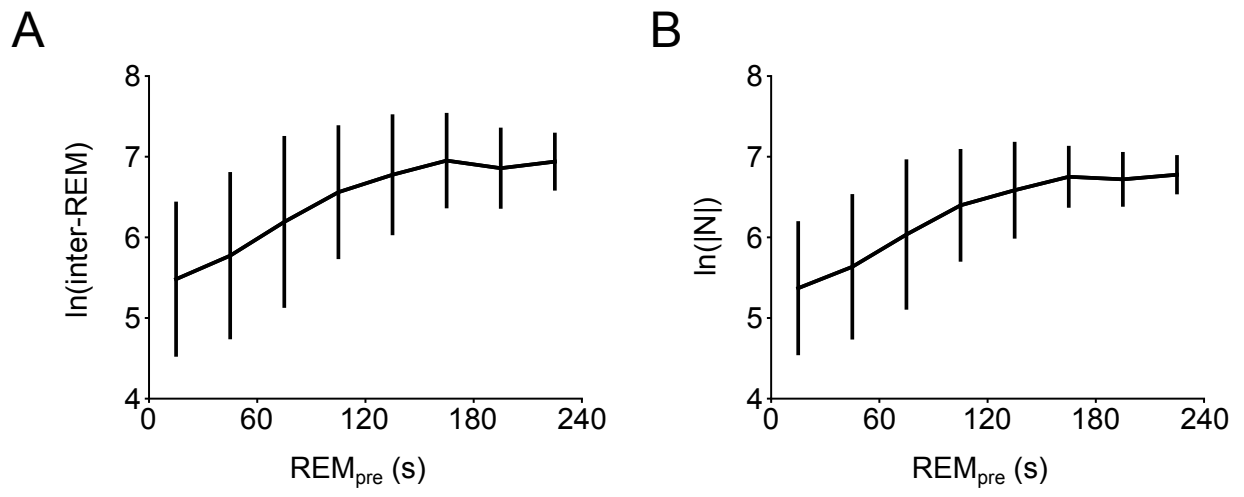

**S2 Fig.** Mean and standard deviation of inter-REM and  $|N|$  as a function of  $\text{REM}_{\text{pre}}$ .

Supplement: S2 Fig — (A) Mean and standard deviation of ln(inter-REM) as a function of REMpre (n = 5090). (B) Mean and standard deviation of ln(|N|) as a function of REMpre. (PDF) [file pcbi.1009316.s002.pdf]

A

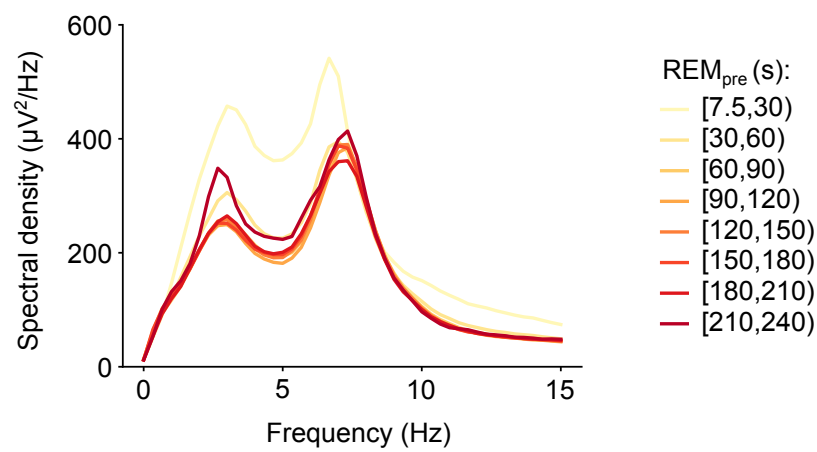

B

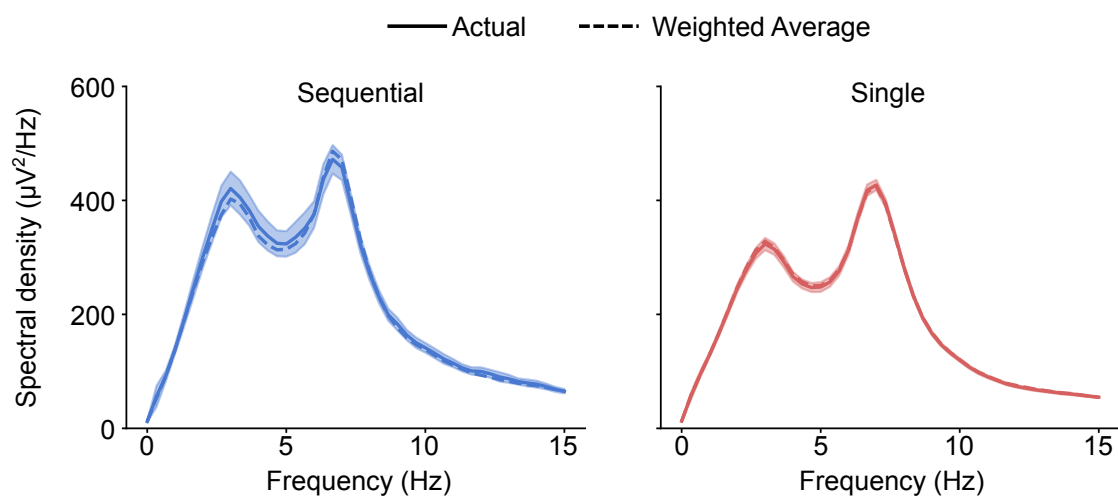

S3 Fig. Prefrontal EEG during REM sleep for sequential and single cycles.

Supplement: S3 Fig — (A) Spectral density of prefrontal EEG during REM sleep for different REM sleep durations (REMpre). (B) Spectral density of prefrontal EEG during REM sleep for sequential and single cycles. Solid lines represent the actual densities with shadings indicating the 99% CIs. The dashed lines represent the weighted averages of the duration-dependent densities in A. The weighted averages were calculated based on the proportion of REMpre values falling into each 30 s bin (Methods). (PDF) [file pcbi.1009316.s003.pdf]

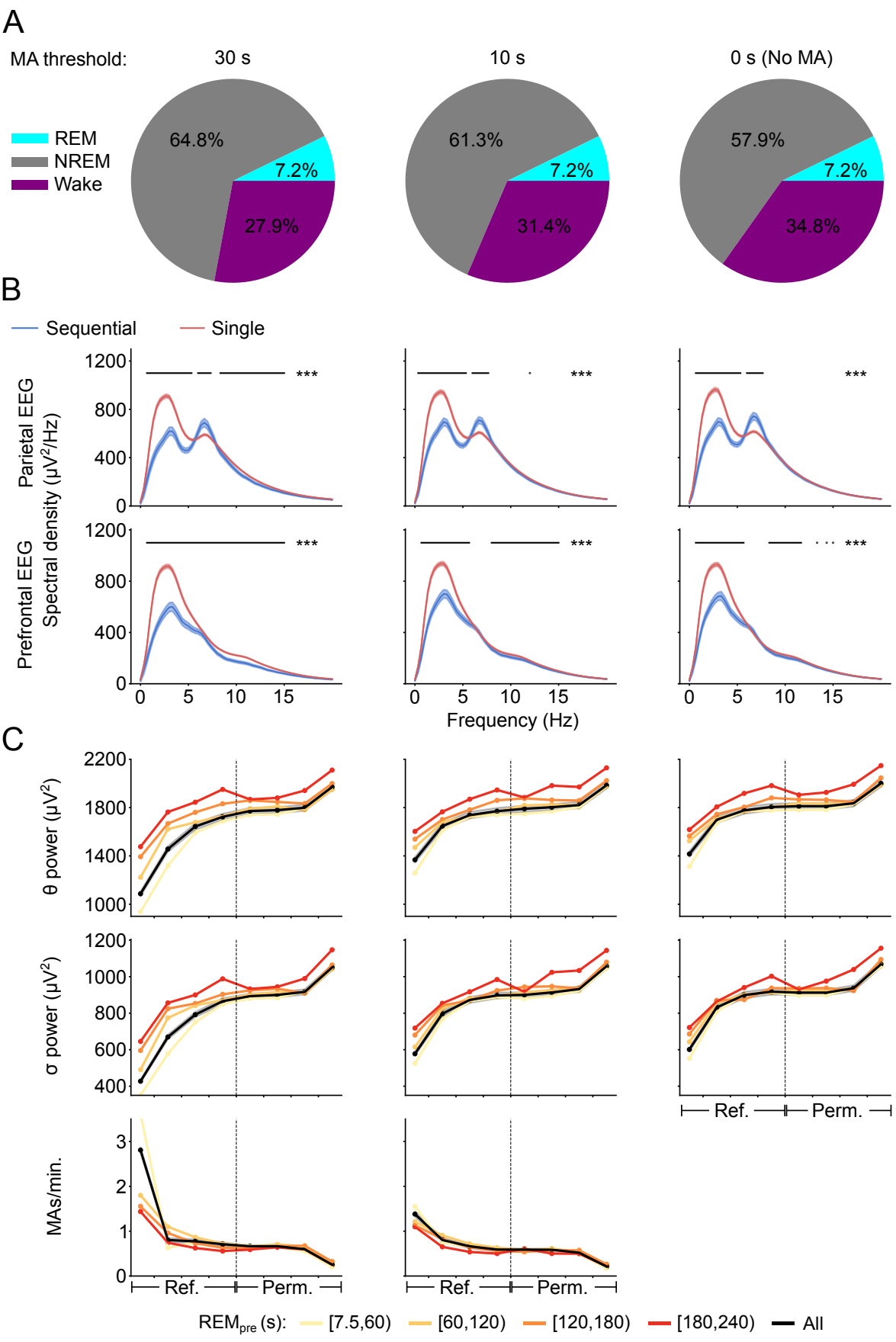

S4 Fig. Comparison of different MA thresholds (1).

Supplement: S4 Fig — (A) Pie chart showing the percentage of REM, NREM, and Wake for different MA thresholds. All wake episodes with duration ≤ 30 s or ≤ 10 s were scored as MA; for the threshold of 0 s, no MAs were scored. (B) Spectral density of parietal (top) and prefrontal EEG (bottom) during NREM sleep for both sequential and single cycles using different MA thresholds. Horizontal lines indicate frequencies at which the spectral densities for sequential and single cycles are statistically different (Welch’s t-test, *** p<0.001). Shadings, 99% CI. (C) Progression of θ power, σ power, and MA rate throughout the refractory and permissive period for different MA thresholds. The durations of both the refractory and permissive period were normalized to unit length and subdivided into quartiles of equal normalized duration. The average for all REMpre values is shown in black. Shadings, 99% CI. (PDF) [file pcbi.1009316.s004.pdf]

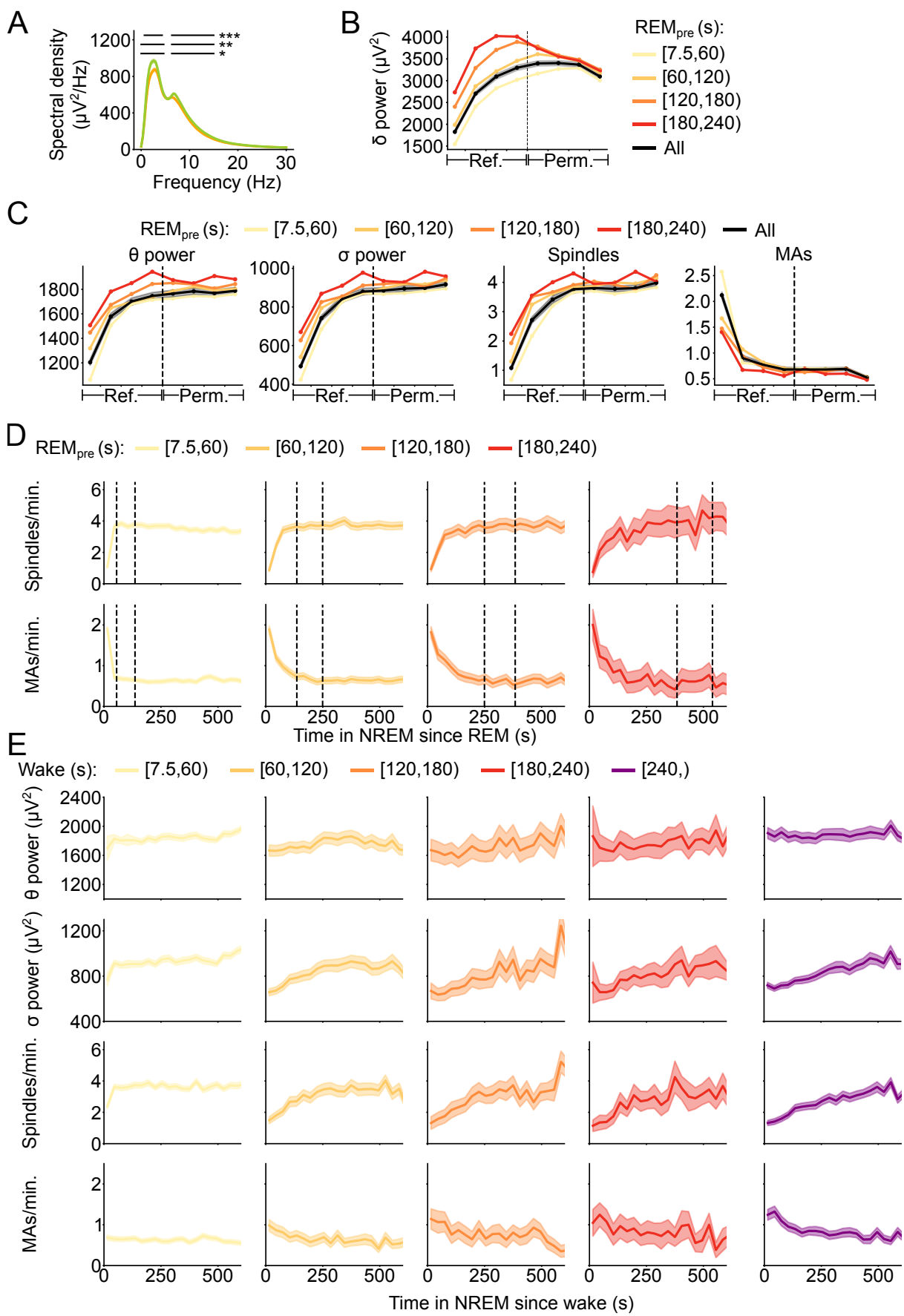

S5 Fig. EEG power, spindle rate, and MA rate throughout the refractory and permissive period.

Supplement: S5 Fig — (A) Spectral density of the parietal EEG for NREM sleep during the refractory and permissive period. Horizontal lines indicate frequencies at which the spectral densities for sequential and single cycles are statistically different; (Welch’s t-test, *** p<0.001, nrefractory = npermissive = 3934). (B) Progression of δ power throughout the refractory and permissive period for different ranges of REMpre. The average for all REMpre values is shown in black. Shadings, 99% CI. (C) Progression of θ power, σ power, rate of spindles, and rate of MAs on normalized time scale throughout NREM sleep. Similar to Fig 4F but with the last 40 s of NREM sleep before the onset of the next REM episode excluded. (D) Progression of spindle rate and MA rate on the non-normalized time scale throughout the first 600 s of NREM sleep during the inter-REM interval for different values of REMpre. The two vertical dashed lines indicate the lowest and highest threshold separating the refractory from the permissive period derived from the low and high bounds of the corresponding REMpre range. Shadings, 99% CI. (E) Progression of θ power, σ power, spindle rate, and MA rate on the non-normalized time scale throughout the first 600 s of NREM sleep after a wake period. Different ranges of wake episode durations were selected to match the corresponding durations of REMpre in D. Shadings, 99% CI. (PDF) [file pcbi.1009316.s005.pdf]

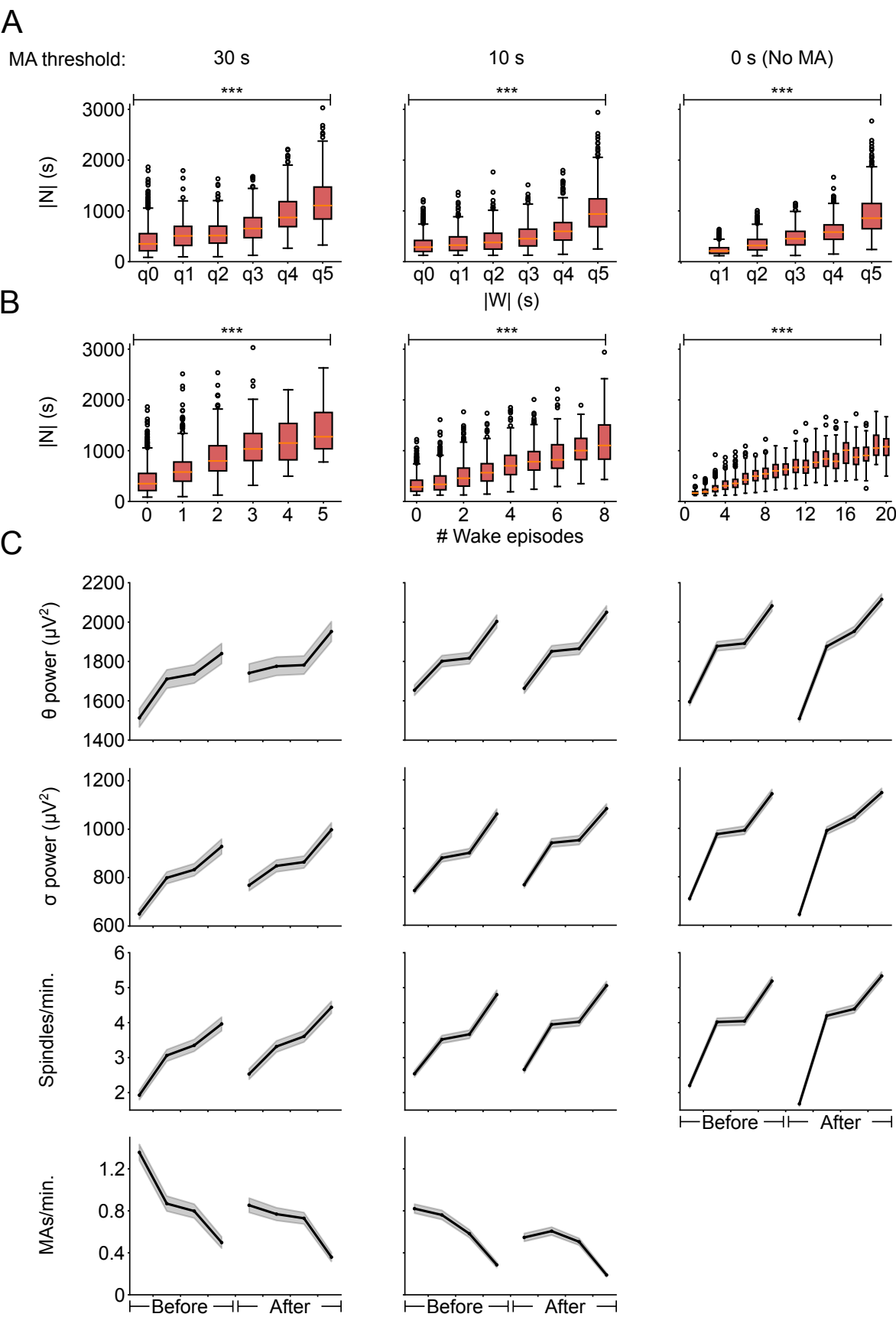

S6 Fig. Comparison of different MA thresholds (2).

Supplement: S6 Fig — (A) Box plots comparing total NREM duration, |N|, for single cycles with increasing values of total wake duration, |W|, for different MA thresholds (30 s: Welch’s ANOVA, F(5,832.43) = 320.67, p = 2.87e-191; 10 s: Welch’s ANOVA, F(5,1850.87) = 359.09, p = 2.22e-269; No MA: Welch’s ANOVA, F(4,1951,53) = 1113.21, p = 0.0). The x-tick q0 corresponds to cycles without wake. The remaining cycles with |W| > 0 were subdivided into quintiles, labeled q1—q5, based on the distribution of |W| for single cycles. For the threshold of 0 s, there was no cycle without wake. (B) Box plots comparing |N| for single cycles based on the number of wake episodes occurring during the inter-REM interval (30 s: Welch’s ANOVA, F(5,249.10) = 281.68, p = 2.54e-100; 10 s: Welch’s ANOVA, F(8,511.64) = 202.86, p = 2.55e-153; No MA: Welch’s ANOVA, F(19,681.55) = 448.18, p = 0.0). (C) Progression of θ power, σ power, spindle rate, and MA rate during NREM sleep before and after a wake episode for different MA thresholds. Only sequences with at least 1 minute of NREM sleep both before and after wake during the inter-REM interval of single cycles were included. The duration of NREM episodes was normalized. ‘Before’ refers to all NREM sleep in between either the previous REM or wake episode and the current wake episode. ‘After’ refers to NREM sleep in between the current wake episode and either the next wake or REM episode. Shadings, 99% CI. (PDF) [file pcbi.1009316.s006.pdf]

A

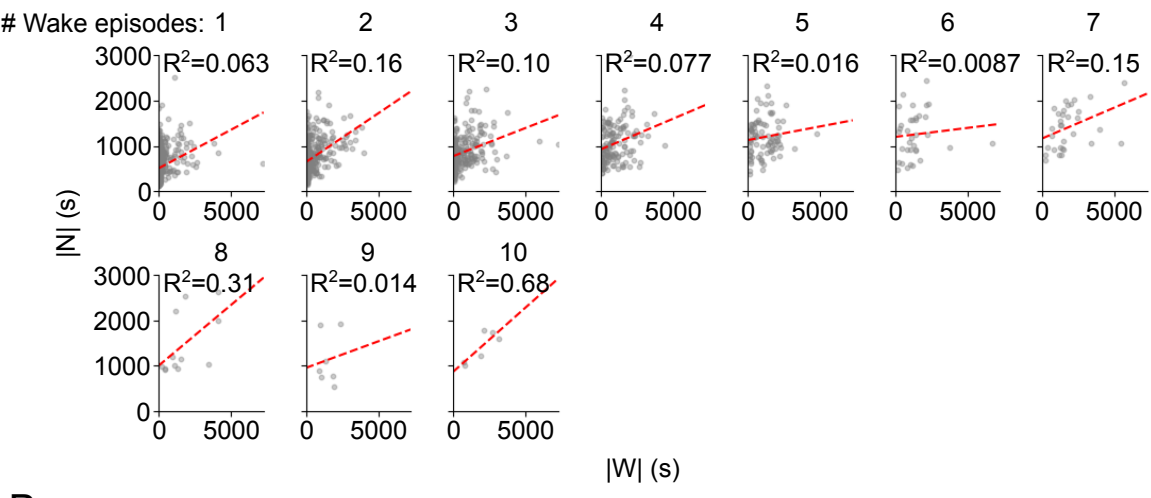

B

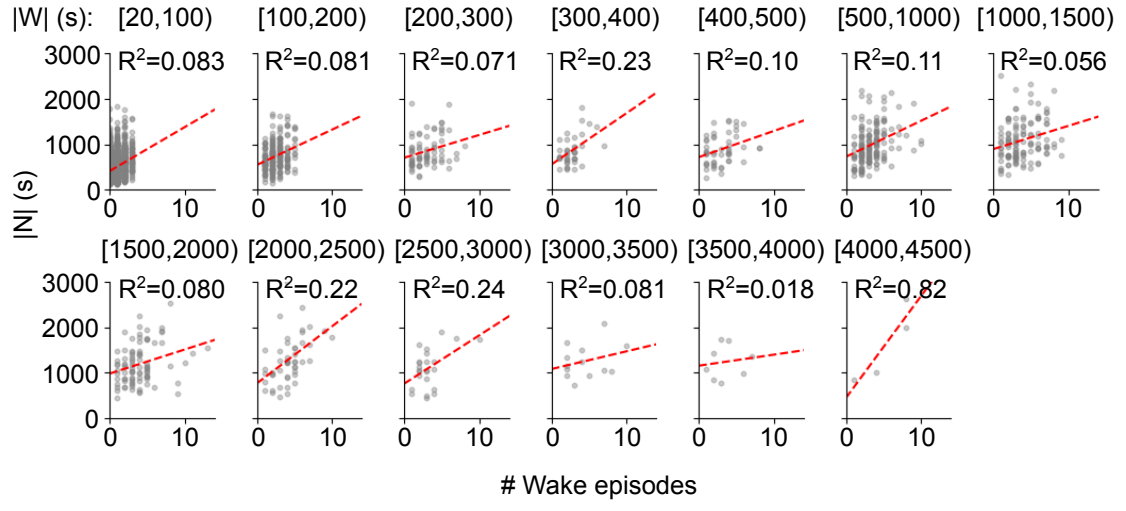

S7 Fig. Relationship between wake episodes and NREM sleep.

Supplement: S7 Fig — (A) Scatter plots for |W| vs. |N| during single cycles for an increasing number (1–10) of wake episodes during the inter-REM interval. For each number of wake episodes, |W| and |N| are positively correlated. Red lines, linear regression fits. (B) Scatter plots for the number of wake episodes vs. |N| during single cycles for increasing ranges of |W|. In each case, the number of wake episodes and |N| was positively correlated. Red lines, linear regression fits. (PDF) [file pcbi.1009316.s007.pdf]

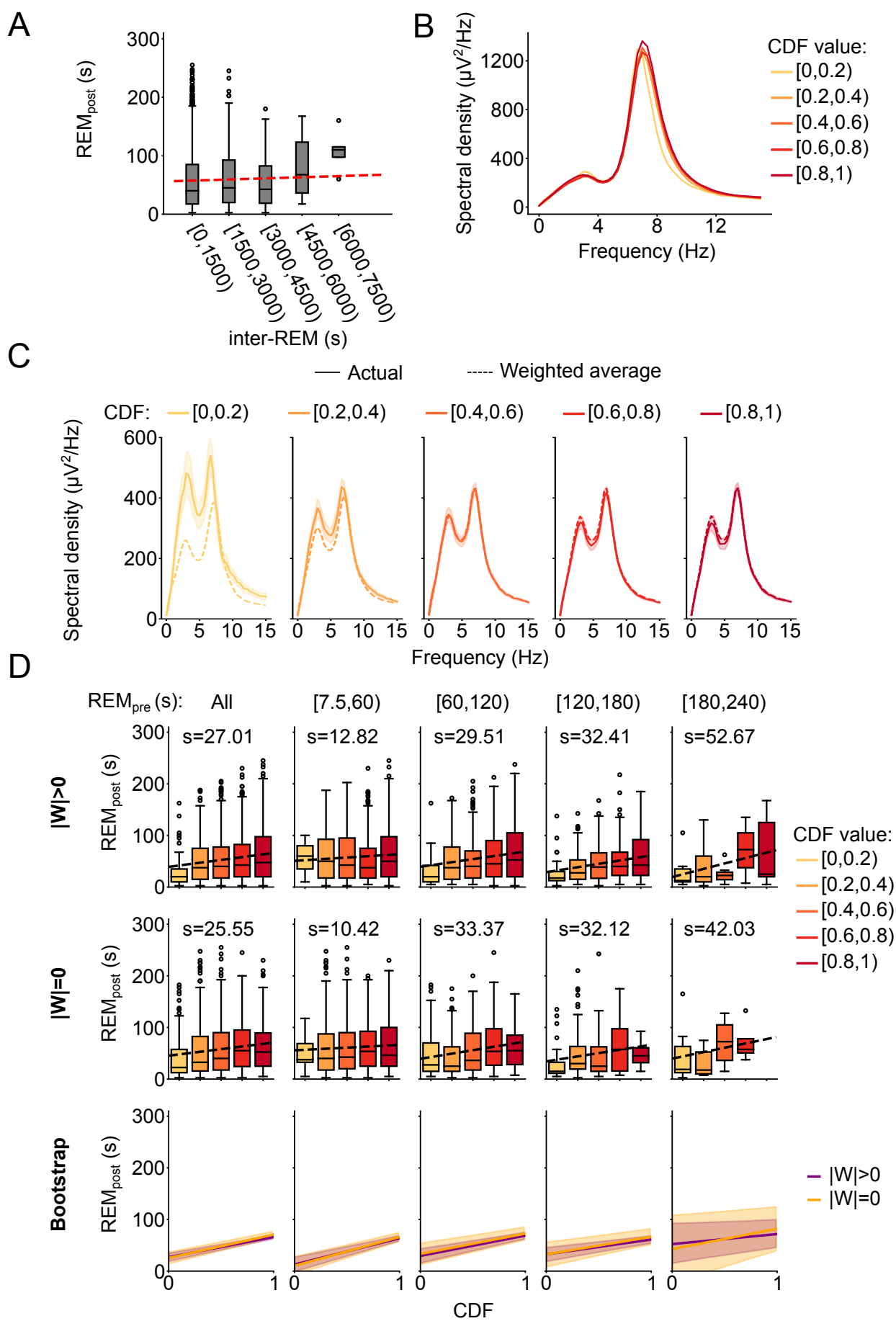

S8 Fig. Variables influencing REM episode duration and EEG.

Supplement: S8 Fig — (A) Box plot comparing the duration of REM sleep (REMpost) following single cycles for different values of inter-REM. Red line, linear regression (slope = 0.0012, R2 = 4.92e-04, p = 0.17). (B) Spectral density of parietal EEG during REM episodes following single cycles for different values of the CDF at REM onset (δ: ANOVA, F(4,3794) = 1.27, p = 0.27; θ: Welch’s ANOVA, F(4,1158.22) = 16.18, p = 6.65e-13; σ: Welch’s ANOVA, F(4,1161.57) = 16.27, p = 5.62e-13). (C) Comparison of the true and estimated spectral densities of REM periods for different ranges of the CDF values at REM onset. Solid lines represent the true spectral densities with shadings representing the 99% CIs. Dashed lines indicate estimated weighted averages (Methods). (D) Comparison of the relationship between the CDF (REM propensity) at REM onset and REMpost for single cycles with wake (|W| > 0) and single cycles without wake (|W| = 0). The first row shows box plots comparing REMpost of single cycles with |W| > 0 with different CDF values at REM onset. The first column shows the correlation for the full range of REMpre; the remaining columns display the results for different bins of REMpre. The second row shows the same comparisons for single cycles with |W| = 0. Dashed lines, linear regression (‘s’ indicates the slope of the regression line). The third row shows the linear regression results of CDF vs. REMpost for single cycles with |W| > 0 and single cycles with |W| = 0. Shadings indicate 95% CIs obtained from 10,000 bootstrap iterations. Columns correspond to different bins of REMpre. (PDF) [file pcbi.1009316.s008.pdf]

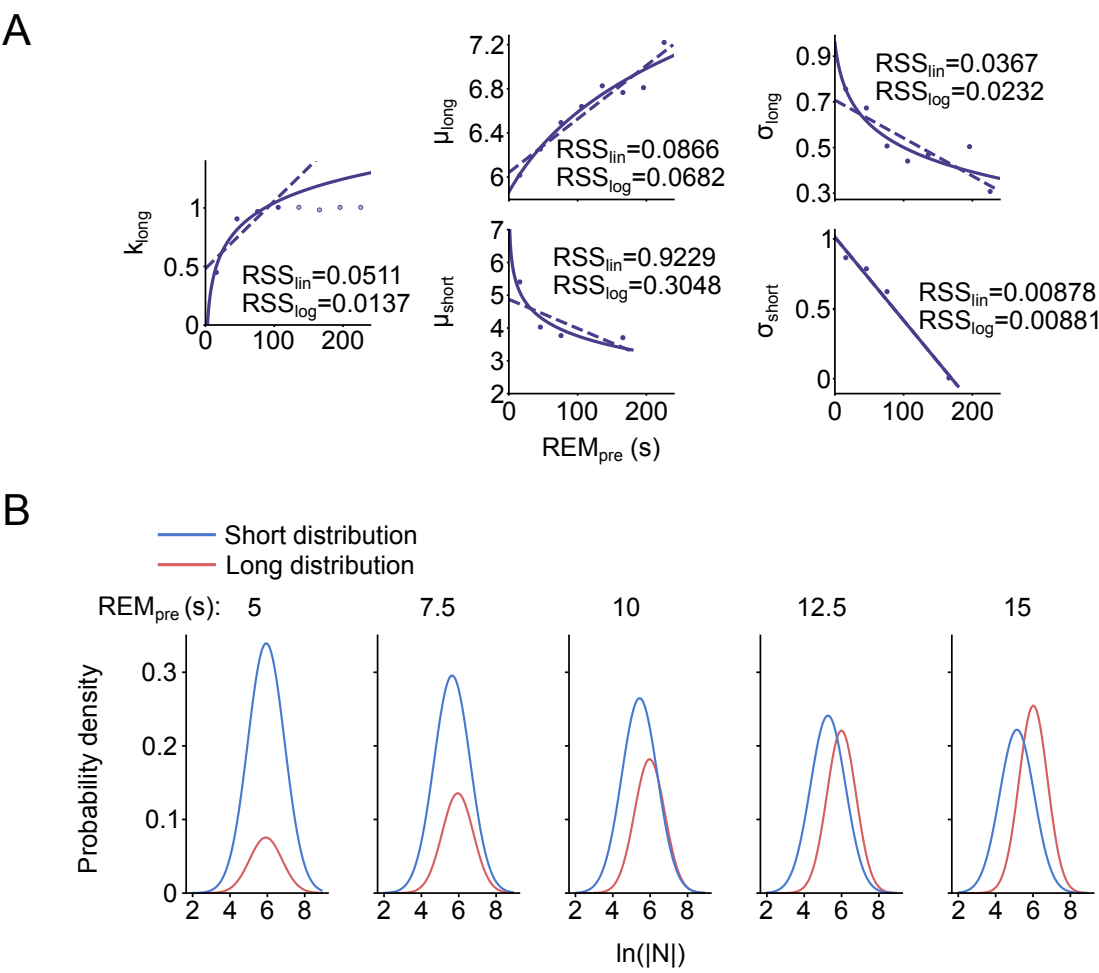

S9 Fig. Conditional GMM for the dark phase.

Supplement: S9 Fig — (A) Comparison of logarithmic and linear fits for the functions describing the relationship between REMpre and each GMM parameter for the dark phase. For klong, the functions were only fitted to the first 4 REMpre splits (filled circles). (B) Estimated PDFs of the short and long Gaussian distributions for each 2.5 s increment of REMpre in the range 5 s—15 s for the dark phase. (PDF) [file pcbi.1009316.s009.pdf]
