# Supplementary material for: A probabilistic model for the ultradian timing of REM sleep in mice
Source: PLoS Comput Biol. 2021 Aug 25;17(8):e1009316. doi: 10.1371/journal.pcbi.1009316 (PMC8423363; doi:10.1371/journal.pcbi.1009316)
Supplement: S1 Table — Mean and standard deviation across animals of key variables in the sleep pattern of mice during the light and dark phase. Note that the R2 values for REMpre vs. |N|, |W|, or inter-REM differ from those in Fig 1B, as linear regression was performed for each animal individually before averaging, instead of computing R2 values for the whole data distribution from all animals. (PDF) [file pcbi.1009316.s010.pdf]

		Light (n = 72 mice)	Dark (n = 35 mice)
Mean state duration (s)	REM	57.20 ± 10.01	63.28 ± 10.36
	$\|N\|$	494.39 ± 124.98	641.13 ± 163.49
	$\|W\|$	196.41 ± 95.43	893.48 ± 307.88
	inter-REM	690.81 ± 200.49	1534.62 ± 417.46
R^2 of REM_{pre} vs.	$\|N\|$	0.29 ± 0.12	0.20 ± 0.15
	$\|W\|$	0.039 ± 0.058	0.035 ± 0.056
	inter-REM	0.13 ± 0.098	0.053 ± 0.074
Mean REM_{pre} duration (s)	Sequential	27.24 ± 8.20	26.06 ± 11.92
	Single	65.71 ± 8.93	73.85 ± 8.30

S1 Table. Inter-individual variability.
