# Supplementary material for: A probabilistic model for the ultradian timing of REM sleep in mice
Source: PLoS Comput Biol. 2021 Aug 25;17(8):e1009316. doi: 10.1371/journal.pcbi.1009316 (PMC8423363; doi:10.1371/journal.pcbi.1009316)
Supplement: S3 Table — The GMM was fitted on the light phase data set for varying thresholds used to score MAs. The columns show the p-values of the Lilliefors-corrected KS-test for the different MA thresholds. (PDF) [file pcbi.1009316.s012.pdf]

REM_{pre}	MA threshold 30 s	20 s	10 s	0 s
[0,30)	0.65	0.61	0.66	0.70
[30,60)	0.94	0.84	0.71	0.80
[60,90)	0.94	0.94	0.87	0.61
[90,120)	0.74	0.74	0.83	0.78
[120,150)	0.87	0.87	0.82	0.88
[150,180)	0.31	0.53	0.51	0.62
[180,210)	0.78	0.77	0.85	0.83
[210,240)	0.77	0.88	0.69	0.38

S3 Table. P-values from Lilliefors-corrected KS-test for GMMs estimated for different MA thresholds.
