# Supplementary material for: A probabilistic model for the ultradian timing of REM sleep in mice
Source: PLoS Comput Biol. 2021 Aug 25;17(8):e1009316. doi: 10.1371/journal.pcbi.1009316 (PMC8423363; doi:10.1371/journal.pcbi.1009316)
Supplement: S4 Table — Each column shows the coefficients (a,b,c) for the logarithmic or linear functions describing each GMM parameter as a function of REMpre (Methods). For all parameters, we used a logarithmic fit except for σshort. (PDF) [file pcbi.1009316.s013.pdf]

k_{long}		μ_{long}		σ_{long}		μ_{short}		σ_{short}	
a	0.17	a	0.62	a	-0.44	a	-0.57	a	-0.0022
b	0	b	27.42	b	61852.61	b	0	b	0.70
c	0.14	c	3.40	c	1538.85	c	6.33	c	N/A

S4 Table. Coefficients of the conditional GMM for the light phase.
