# Supplementary material for: A probabilistic model for the ultradian timing of REM sleep in mice
Source: PLoS Comput Biol. 2021 Aug 25;17(8):e1009316. doi: 10.1371/journal.pcbi.1009316 (PMC8423363; doi:10.1371/journal.pcbi.1009316)
Supplement: S5 Table — The columns indicate for each range of REMpre the number of sleep cycles, the weight of the long Gaussian distribution, mean and standard deviation of the short and long Gaussian distribution and the p-value obtained for the Lilliefors-corrected KS test for the dark phase (Methods). (PDF) [file pcbi.1009316.s014.pdf]

REM_{pre}	n	k_{long}	μ_{long}	σ_{long}	μ_{short}	σ_{short}	p
[0,30)	350	0.44	6.012	0.75	5.38	0.86	0.81
[30,60)	302	0.90	6.24	0.67	4.01	0.78	0.94
[60,90)	229	0.96	6.48	0.50	3.75	0.61	0.77
[90,120)	186	1	6.63	0.43	N/A	N/A	0.68
[120,150)	118	1	6.82	0.46	N/A	N/A	0.25
[150,180)	45	0.97	6.76	0.42	3.68	0.0010	0.46
[180,210)	6	1	6.80	0.50	N/A	N/A	0.96
[210,240)	6	1	7.21	0.30	N/A	N/A	0.87

S5 Table. GMM parameters for the dark phase.
