# Supplementary material for: A probabilistic model for the ultradian timing of REM sleep in mice
Source: PLoS Comput Biol. 2021 Aug 25;17(8):e1009316. doi: 10.1371/journal.pcbi.1009316 (PMC8423363; doi:10.1371/journal.pcbi.1009316)
Supplement: S6 Table — Each column shows the coefficients (a,b,c) for the logarithmic or linear functions describing each GMM parameter as a function of REMpre for the dark phase (Methods). For all parameters, we used a logarithmic fit except for σshort. (PDF) [file pcbi.1009316.s015.pdf]

k_{long}		μ_{long}		σ_{long}		μ_{short}		σ_{short}	
a	0.29	a	0.89	a	-0.16	a	-0.72	a	-0.0059
b	0	b	80.23	b	6.30	b	0	b	1.012
c	-0.30	c	1.94	c	1.25	c	7.11	c	N/A

S6 Table. Coefficients of the conditional GMM for the dark phase.
