# Supplementary material for: A probabilistic model for the ultradian timing of REM sleep in mice
Source: PLoS Comput Biol. 2021 Aug 25;17(8):e1009316. doi: 10.1371/journal.pcbi.1009316 (PMC8423363; doi:10.1371/journal.pcbi.1009316)
Supplement: Attachment — Submitted filename: Response_to_reviewers.pdf [file pcbi.1009316.s017.pdf]

Reviewer #1 (*reviewer's comments in italic*):

This paper addresses an important unsolved problem in sleep research: the genesis of REM/NREM sleep cycles. The authors find that the dynamics in mice can be adequately explained in terms of a bifurcation between sequential REM bouts (i.e., essentially a continuation of the REM state) and a refractory period that separates distinct REM bouts, with the length of this period being dependent on the length of the preceding REM bout. The paper provides a powerful treatment of this issue. I applaud the authors for being so incredibly thorough with their analysis of the data. This approach allows a number of alternate interpretations to be directly tested. Overall I enjoyed reading the paper. Please see my suggestions below.

We thank the reviewer for these supportive comments!

1) Regarding the sequential bouts, the authors muse on the idea that the interceding NREM bouts appear to have some features of REM and thus may be a sort of intermediate state. Have the authors considered this pattern in the context of local sleep?

In rodents, REM sleep is preceded by an intermediate state (also called NREM to REM transition state) lasting on average about 20 s. This stage bears resemblance to local sleep given that different parts of the brain (e.g. the hippocampus) may have already transitioned to REM sleep, whereas others are still in NREM sleep at the same time (Durán et al., 2018; Gottesmann, 1996). Similarly, sleep during sequential sleep cycles may be local in the sense that at the same time different brain regions may reside in different states, giving rise to an average EEG with features of both NREM and REM sleep.

For the automatic sleep classification and the following manual rescoring we only used the parietal EEG. Based on the annotation using a single EEG channel we cannot judge whether sequential NREM sleep bears features of local sleep. As we mention in the revised Discussion (page 21, lines 623 - 628), the best way to test this interesting idea would be to simultaneously record local field potentials from multiple sites to determine whether the activity in different brain areas shows signs of different, simultaneously existing local brain states.

2) The genesis of REM/NREM sleep cycles has been considered previously using dynamical systems models. These models have helped to propose and test potential theoretical mechanisms. Specifically, see:

Diniz Behn CG, Ananthasubramaniam A, Booth V. Contrasting existence and robustness of rem/non-rem cycling in physiologically based models of rem sleep regulatory networks. SIAM Journal on Applied Dynamical Systems. 2013;12(1):279-314.

Phillips AJ, Robinson PA, Klerman EB. Arousal state feedback as a potential physiological generator of the ultradian REM/NREM sleep cycle. Journal of theoretical biology. 2013 Feb 21;319:75-87.

It would be valuable to discuss the present findings in the context of these candidate models. I can imagine that the sequential states could be readily explained within a dynamical model by introducing stochasticity, as this could cause temporary transit between REM and NREM sleep, even before a REM sleep bout has really concluded. The positive association between REMpre and N is clearly in line with models that assume a homeostatic balance of the stages (and probably not in line with models that assume a clock-like mechanism). However, it is less clear to me whether the findings for W are consistent with existing dynamical models, or whether models would need to be amended to explain these results.

We fully agree with the reviewer that it would be very informative to test to what extent existing dynamical systems models can reproduce our observations and to explore what changes or additions (such as noise terms) are required to capture the described statistics of sleep cycles. We now discuss such models in the context of sequential sleep cycles (page 22, lines 629 - 639).

3) I would avoid using the word "impact" in cases where you do not establish causality or direction of effects (which is not established simply by temporal precedence), such as on line 137.

As recommended by the reviewer, we avoid in the revised manuscript the word ‘impact’ (as it may imply causality) throughout the text. More specifically,

- we replaced “To systematically describe the impact of REM sleep on the duration and quality of subsequent NREM sleep” with “To systematically describe the interaction between REM sleep and the duration and quality of subsequent NREM sleep” (page 6, lines 142 - 144).
- We replaced the heading “Impact of wakefulness on NREM sleep” with “Relationship between wakefulness and NREM sleep” (page 14, line 395).
- We replaced the heading of Figure 5 “Impact of wake episodes on NREM sleep during an inter-REM interval.” with “Relationship between wake episodes and NREM sleep during an inter-REM interval.”
- We replaced the heading of S11 Figure “Impact of wake episodes on NREM sleep” with “Relationship between wake episodes and NREM sleep” (page 44, line 1457).
- In the Discussion, we have substituted the heading “Impact of REM propensity on REM duration and EEG” with “Interaction between REM propensity and REM episode duration” (page 23, line 676).
- In the Author Summary, we replaced “to understand how REM sleep impacts the quality of future NREM sleep throughout the sleep cycle” with “to investigate the relationship between REM sleep and the quality of future NREM sleep” (page 3, lines 48 - 49).
- We only left the term ‘impact’ in the text if it was preceded by “suggest that” or “indicate that” to make sure that the discussed findings merely indicate or suggest an “impact” rather than proving it.

4) I think that the treatment of REMpre<30 s requires some more consideration/justification. In the text, the authors say that "we found that for values of REMpre < 150 s,142 ln(|N|) forms a bimodal distribution". But this is clearly not the case for REMpre < 30 s, so this statement is not accurate. The argument for effectively treating this as bimodal on Line 723 is not strong. A distribution can be unimodal without being Gaussian, as appears to be the case here. A decision on the basis of the Shapiro-Wilk test therefore does not make sense. My interpretation looking at Fig. 2E is that the unimodality for short REM bouts is effectively due to blending of the two types of NREM bouts (short and long), which would arguably justify treating it as a bimodal distribution.

We now explicitly state in the Results that "for values of REM_{pre} in the range from 30 s to 150 s, ln(|N|) forms a bimodal distribution" (page 6, line 147). In the Methods, we now state that we assume that the unimodality of the distribution in the range from 0 s to 30 s results from the blending of the distributions for short and long cycles (page 28, lines 801 - 803).

5) Line 752: "We calculated the residual sum of squares (RSS) forlong753 both the linear and logarithmic fits and chose the function for which the RSS was lower." - This is a 2-parameter vs. 3-parameter fit comparison. Since the slope of the logarithmic function is a/(x+b)+c, it can also be made to approximate a linear fit for very large |b|. Without bounds on a, b, c, this does not seem to be a meaningful comparison (i.e., the log function is theoretically guaranteed to do better), as the logarithmic function could always be made to fit the data at least as well as the linear fit based on RSS. The comparison would make sense only with adjustment for the number of parameters (e.g., with AIC). It may be simplest to just drop the linear fits.

While revisiting the exact fitting process, we realized that we had mistakenly stated that "fitting was performed using the Levenberg-Marquardt algorithm implemented in scipy" (line 754 of the previous version). In truth, we had constrained the parameter b in a*ln(x+b)+c to the ranges [0,inf) because the log function is not defined for negative values and we used the Trust Region Reflective algorithm implemented in scipy. We apologize for this mistake. We have corrected the description in the revised Methods (page 29, lines 827 - 836).

As explained by the reviewer, for arbitrarily large values of |b| the logarithmic function is expected to perform at least as well as the linear function. But for the case that the data is (close to) truly linear, a linear fit may outperform the log fit as the log function is only approximately linear. For example in the case of σ_{low}, the estimated linear model indeed outperforms the logarithmic model in terms of RSS (**S5 Figure A**). The linear fit also outperforms the log fit for σ_{low} when comparing the two functions using AIC. Although the linear and log fit are arguably very similar, and, as suggested by the reviewer, we could therefore simply drop the linear fit, we would argue that one benefit of keeping it is to slightly reduce the number of model parameters.

References

Durán, E., Oyanedel, C.N., Niethard, N., Inostroza, M., and Born, J. (2018). Sleep stage dynamics in neocortex and hippocampus. Sleep *41*.

Franken, P. (2002). Long-term vs. short-term processes regulating REM sleep. J. Sleep Res. *11*, 17–28.

Gottesmann, C. (1996). The transition from slow-wave sleep to paradoxical sleep: evolving facts and concepts of the neurophysiological processes underlying the intermediate stage of sleep. Neurosci. Biobehav. Rev. *20*, 367–387.

Reviewer #2 (*reviewer's comments in italic*):

I am really pleased to see this strong support for the ideas we advanced many years ago – your reference 19. I cannot offer any expertise in the review of your mathematical modeling, but I can raise questions and suggestions that you might find useful to consider.

We thank the reviewer for this important discussion comparing the results of our study with those in Benington et al. 1994. As we will argue below, while there are differences in the details of the data interpretation, our results provide further support for the key findings by Benington et al. 1994, rather than contradicting them.

First, there is an important difference between your basic approach to the EEG analysis and ours, and this difference could be at the root of some of our apparent conflicts in interpretation. Please check our methods paper, Benington, Kodali, Heller (1994) Scoring transitions to REM sleep in rats based on the EEG phenomena of Pre-REM sleep: an improved analysis of sleep structure. Sleep 17(1):28-36. We both used spectral analysis of key frequency bands, but the important difference is that we used those signatures to identify transitions to REM even when no scorable REM ensued. I believe you used a scoring threshold of 30 sec of scorable REM, but maybe I am wrong. These NRTs, as we called them, are an excellent indicator of REM pressure.

First, we have to clarify a misunderstanding: We used 2.5 s epochs for sleep scoring and we did not apply any threshold requiring a minimum duration of REM sleep. In other words, all our analyses include REM periods < 30 s. Given that the distribution of REM sleep durations in mice is unimodal, we see no stringent statistical criterion for separating short from long REM sleep episodes (**Reviewer Figure 2.1**). According to our definition, a sleep cycle ranges from the start of the preceding REM sleep period (of any duration) till the beginning of the next REM sleep period (of any duration).

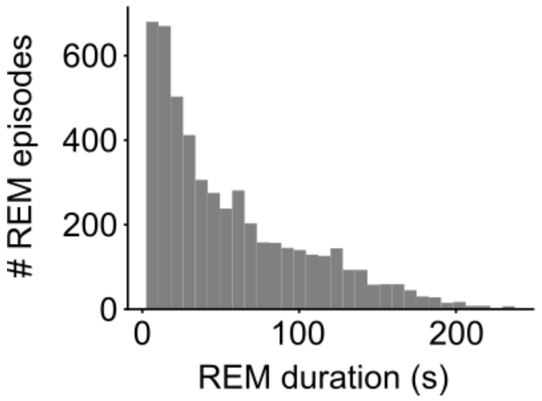

Reviewer Figure 2.1. Distribution of REM sleep episode durations. Histogram of REM sleep durations recorded in our data set from n = 72 mice.

Here are some comments based on figures in your Ref 19. First, if you compare the distribution of NREM episode durations using only REM episodes > than 30 sec, vs. looking at the durations of NREM episodes as a function of all inter NRT intervals, you get very different distributions. I think that the latter analysis yields a more likely representation of a homeostatic system.

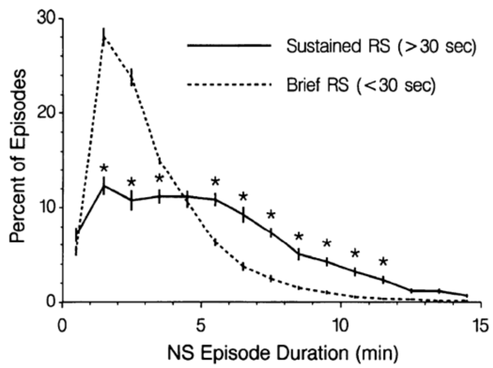

Bennington et al. 1994 - Figure 3

As we stressed above, our analyses include REM sleep episodes < 30 s and there is no major difference in the scoring of REM sleep between Benington et al. 1994 and our study. In fact, when we plot our data from mice the same way as in Fig 3 in Benington et al. 1994, we similarly find that short REM sleep episodes (< 30 s) are followed by a shorter total duration of NREM sleep (**Reviewer Fig. 2.2**).

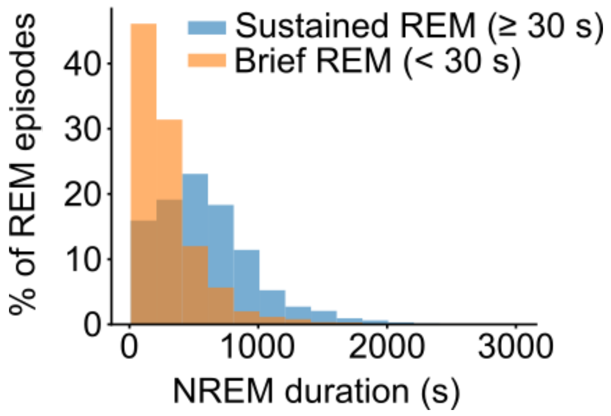

Reviewer Figure 2.2. NREM sleep episode duration distribution for NREM sleep episodes after sustained vs. brief REM sleep episodes.

Second, if you use the NRT scoring for the brief REM sleep episodes, you see a beautiful relationship indicating the build up of REM pressure.

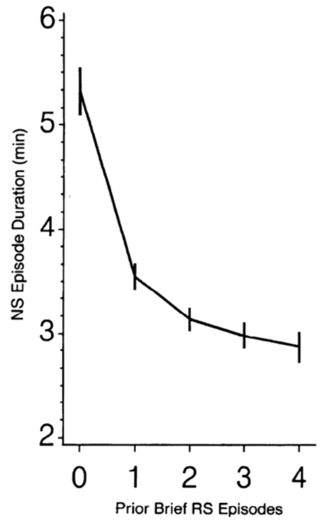

Bennington et al. 1994 - Figure 4

We believe that one major difference between our study and Benington et al. 1994 is in the interpretation of short REM sleep episodes (< 30 s). Benington et al. 1994 uses their occurrence as an indicator of REM sleep pressure, while we do not distinguish them from longer REM sleep periods. In other words, we treat REM episodes < 30 s exactly the same as REM episodes > 30 s. Our rationale for not distinguishing short from long REM episodes is that the distribution of REM sleep episodes in mice is unimodal (**Reviewer Figure 2.1**) and we therefore did not find a clear statistical criterion (threshold) separating short from long REM sleep episodes. Instead of using the frequency of short REM sleep periods as a measure for REM pressure, we estimated the REM propensity using the cumulative distribution function (CDF) of our model. We found that the REM propensity at REM sleep onset is positively correlated with the duration of REM sleep episodes (**Fig 6C**); that is, entering REM sleep with higher propensity results in longer REM sleep episodes. The strength of the correlation is the same, irrespective of whether the preceding sleep cycle does or does not contain wake bouts (**S11 Figure D** in the revised manuscript). This finding provides further evidence for the notion that REM sleep is regulated by a homeostatic process accumulating during NREM sleep. Thus, our paper supports and does not contradict this key finding by Benington et al. 1994 as we now explicitly state in the revised Discussion (page 24, lines 729 - 731).

Third, you postulate the existence of a "refractory period" with no evidence for such a phenomenon other than the data based on your rules of analysis. If you use NRTs rather than sustained REM episodes, there is no indication of a refractory period. Consider the following data also from ref. 19. The probability of a brief REM episode as determined by an NRT is the same no matter whether the sleep cycle has just begun (ie, no refractory period), is midway, or is nearing completion.

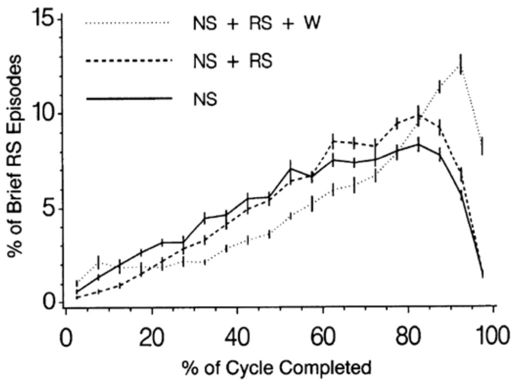

Bennington et al. 1994 - Figure 7

The major concern raised by the reviewer is about our claim of the existence of a refractory period. **Fig 1B** middle and **Fig 2A** show in a scatter plot the correlation between the duration of the preceding REM sleep episode (REM_{pre}) and the total duration of NREM sleep (|N|) during the following sleep cycle (**Fig 2A** is identical to **Fig 1B** but with a logarithmic y-axis). Importantly, these plots include every REM sleep episode without any restriction on their duration. They are thus identical to what is shown in **Fig 2** of Benington et al. 1994 for rats, except that we plot every single data point (corresponding to a REM period and the following total NREM sleep duration).

We argue that two features become apparent in this scatter plot: (1) The distribution of REM_{pre} vs |N| is bimodal: There is a tail of data points along the x-axis and on top of it, an elongated cloud of data points for which |N| increases with REM_{pre}. (2) Particularly visible for large REM_{pre} values, the upper distribution is preceded by an interval of NREM sleep during which the occurrence of REM sleep is highly unlikely (and which we refer to as the refractory period). In other words, especially after a long REM episode, it is very unlikely that the animal will immediately re-enter REM sleep. One major rationale to fit a lognormal mixture model was to define a formal criterion to separate the two distributions. We illustrate these 2 points in **Reviewer Figure 2.3**.

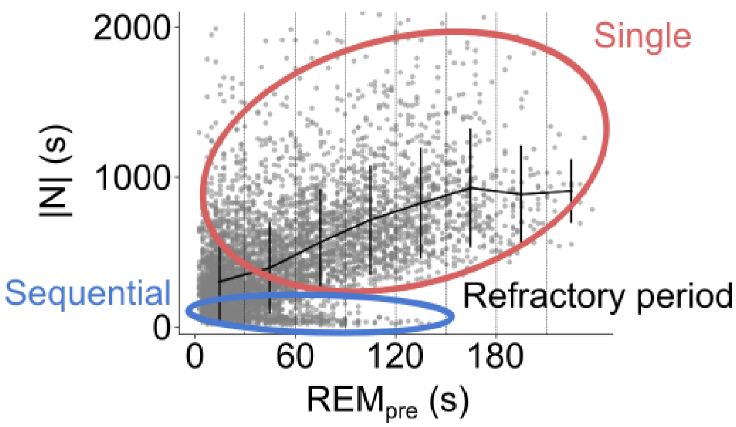

Reviewer Figure 2.3. Scatter plot of REM_{pre} vs. |N|. Each dot shows the duration of the preceding REM episode (REM_{pre}) and the following total duration of NREM sleep (|N|) till the next REM episode. The solid line and error bars show the average NREM duration and standard deviation for consecutive 30 s bins of REM_{pre}.

Based on **Fig 7** of Benington et al. 1994, the reviewer argues that there is no evidence for a refractory period following a REM sleep episode: The percentage of brief REM periods continuously increases throughout the sleep cycle without any indication of an interval during which the probability to enter REM is close to zero. To construct **Fig 7** in Benington et al. 1994, the authors included all cycles with the preceding REM episode lasting > 30 s. We performed the same analysis and found that on the aggregate, the results are the same in our data set from mice ("All" in **Reviewer Figure 2.4**). However, when we look deeper into the data by splitting the cycles based on the duration of REM_{pre}, we find two crucial points: (1) For cycles with REM_{pre} in the range 60 s ≤ REM_{pre} < 150 s, there is a small peak in the probability of entering of brief REM sleep episode at the beginning of the cycle. This corresponds to sequential cycles. (2) For cycles with REM_{pre} ≥ 150 s, there is 0 probability to enter a brief REM sleep episode at the initial portions of the cycle. This is what we claim to be the refractory period. REM sleep is followed by an interval of NREM sleep during which it is highly unlikely to enter the next REM episode and this interval increases in duration with the duration of REM_{pre}.

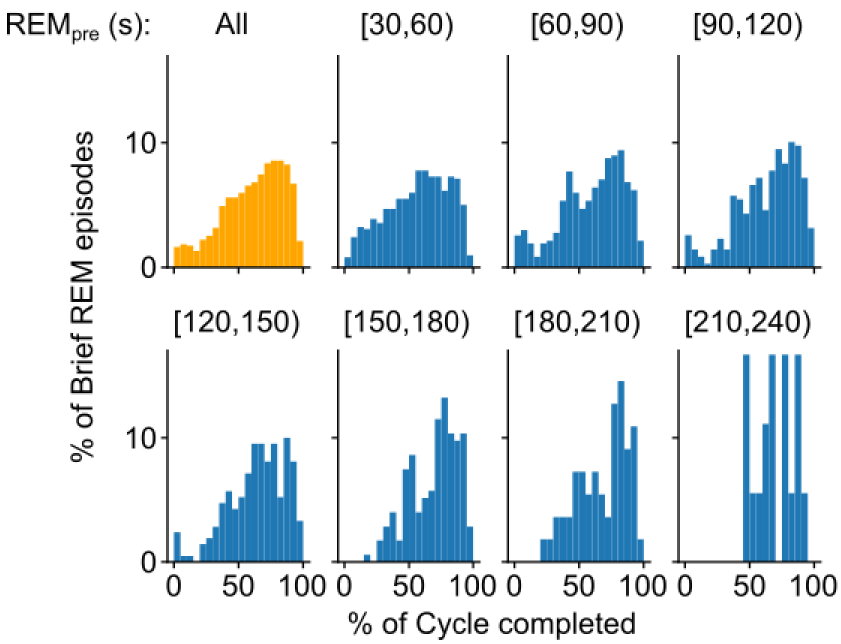

Reviewer Figure 2.4. Timing of brief REM sleep episodes (< 30 s) as a function of relative position within the sleep cycle. Sleep cycles were defined as in **Fig 7** of Benington et al. 1994: A sleep cycle ranges from the start of the preceding REM episode with duration > 30 s till the beginning of the next REM episode with duration > 30 s. The duration of sleep cycles was normalized to range from 0 to 100% and includes only NREM sleep. “All” (orange) includes all sleep cycles preceded by a REM sleep episode with duration REM_{pre} > 30 s (as **Fig 7** in Benington et al. 1994). The following (blue) plots show the same analysis but restricted to cycles preceded by REM episodes with durations (REM_{pre}) within the range indicated on top of each plot.

I know this is an old paper and does not make use of sophisticated modeling, but I think the data are reflecting the true nature of REM – NREM interactions more precisely than the analysis you have used that has led you into proposing a refractory period for which there is no evidence.

Instead of contradicting the findings by Benington et al. 1994, we strongly believe that we extended the framework provided by Benington et al. by explicitly modeling the full distribution of REM_{pre} vs. following NREM sleep duration. The bimodal nature of this distribution led us to distinguish between single and sequential sleep cycles. We then separated single sleep cycles into a refractory and permissive period allowing us to study how electrophysiological features change with the propensity for REM sleep.

Comments specific to text.

Starting on line 80. A lot rides on this introduction of the concept of refractory period which you acknowledge is not defined, nor is there any evidence. The results cited fit perfectly well with a

homeostatic model – a long REM episode more fully discharges the need for REM and is therefore permissive of NREM.

The statistical analysis of the full data distribution (REM_{pre} vs subsequent duration of NREM sleep, |N|) led us to the concept of the refractory period. Given that the refractory period is a major focus of our study, we wanted to introduce and acknowledge in the introduction previous studies/reviews that proposed this notion. Our specific contribution was to introduce a formal statistical definition of the refractory period and to identify features in the EEG, spindle and microarousal rate characteristic for this interval.

Line 104. All REM-NREM cycles fit on the same relationship, so why divide them into two groups?
Lines 211-215. I disagree.

(Line 104 and lines 211-215 both refer to the separation of sleep cycles into single and sequential cycles.)
As mentioned above, plotting the duration of the preceding REM episode (REM_{pre}) vs. the following total NREM duration (|N|) in a scatter plot shows a clear bimodal distribution with short cycles in a long tail along the x-axis and long cycles, the duration of which increases with REM_{pre} (**Reviewer Figure 2.3**). The bimodality of the distribution led us to separate sleep cycles into two groups. The bimodality of the inter-REM distribution has also been reported in rats (Amici et al., 1994; Bassi et al., 2009; Gregory and Cabeza, 2002), cats (Ursin, 1970), monkeys (Kripke et al., 1968) and humans (Carskadon and Rechtschaffen, 2000; Merica and Gaillard, 1991).

Lines 274-276. This is to be expected because at the transition to NREM, it takes about a minute to reach full delta power. If unsuccessful attempts to enter REM occur frequently, the consequence will be lower delta power.

In single cycles, both delta and theta power are reduced following REM sleep (**Fig 4F,G, S9 Figure B**). While the delta power is also reduced during sequential cycles, the theta power is increased (**Fig 3F**). We therefore believe that the EEG during sequential cycles is more similar to that during the intermediate stage preceding transitions to REM sleep as mentioned on page 11, lines 286 - 291.

Without knowing what the trigger for REM sleep transition is, it is not possible to experimentally test the concept of a refractory period. However, you show nicely that the REM-NREM relationship is homeostatic, and the data can be interpreted more parsimoniously by that homeostatic relationship. The problem is that you have set an arbitrary definition of a REM episode rather than recognize the existence of a transition to REM in the absence of that arbitrary value.

As we have stressed above, we do not distinguish between short and long REM sleep periods. The refractory period is clearly visible in the full data distribution (**Reviewer Figure 2.3)**, it is statistically well defined and it is therefore not the mere result of a sophisticated modeling approach.

You have great data, but because I do not feel qualified to evaluate your modeling, and because you might consider my major criticism just a defense of another view point, I am submitting the review without a recommendation, but I hope you will take my comments into consideration.

Best,
Craig Heller

References

Amici, R., Zamboni, G., Perez, E., Jones, C.A., Toni, I., Culin, F., and Parmeggiani, P.L. (1994). Pattern of desynchronized sleep during deprivation and recovery induced in the rat by changes in ambient temperature. J. Sleep Res. *3*, 250–256.

Bassi, A., Vivaldi, E.A., and Ocampo-Garcés, A. (2009). The time course of the probability of transition into and out of REM sleep. Sleep *32*, 655–669.

Carskadon, M., and Rechtschaffen, A. (2000). Monitoring and staging human sleep. In Principles and Practice of Sleep Medicine, (Philadelphia: W.B. Saunders), pp. 1197–1215.

Gregory, G.G., and Cabeza, R. (2002). A Two-State Stochastic Model of REM Sleep Architecture in the Rat. J. Neurophysiol. *88*, 2589–2597.

Kripke, D.F., Reite, M.L., Pegram, G.V., Stephens, L.M., and Lewis, O.F. (1968). Nocturnal sleep in rhesus monkeys. Electroencephalogr. Clin. Neurophysiol. *24*, 581–586.

Ursin, R. (1970). Sleep stage relations within the sleep cycles of the cat. Brain Res. *20*, 91–97.
Watson, B.O., Levenstein, D., Greene, J.P., Gelinas, J.N., and Buzsáki, G. (2016). Network Homeostasis and State Dynamics of Neocortical Sleep. Neuron *90*, 839–852.

Merica, H., and Gaillard, J.-M. (1991). A study of the interrupted REM episode. Physiol. Behav. *50*, 1153–1159.

Reviewer #3 (***reviewer's comments in italic***):

This manuscript is an important and stimulating contribution to the understanding of the ultradian organization of sleep that gives further support to notion that hourglass processes may significantly contribute to shape the architecture of the sleep –wake cycle. It also recognizes the centrality of REM sleep episodes (and its associated hourglass mechanism) in the timing of ultradian sleep cycles. The analytic strategy is elegant, solid and highly reliable, and is an invitation to be replicated in other animal models including humans. This work also confirms in mice the existence of two separate categories of REM sleep episodes (sequential and single) that were described by the Bologna group more than twenty years ago (Amici et al 1994). It also confirms that the factor "time of day" (see below) affects the timing of the REMs hourglass process (as described in ref 24: Vivaldi et al. 2005).

In my opinion, the main theoretical contribution the authors are: (i) incorporates the recently proposed REMs dependent "refractory period" as a relevant timing mechanism within the ultradian cycle of mice as has been described in rats; (ii) that the REMs "refractory period" is an exclusive attribute of single REMs episodes.; (iii) that REMs episodes duration may be determined within ultradian cycles by REMs propensity estimated by the Cumulative Density Function of NREM.

We thank the reviewer for the thorough assessment of our findings and the insightful feedback on the interpretation of our findings.

I have the following observations:

1) The definition of REM sleep propensity as the CDF is clear but the relationship between N an W in the buildup of REM sleep propensity is in my opinion not clearly stated. It is a complicated issue because it could be discussed form the point of view of the relationship between REMpre vs. inter-REMinterval, and between inter-REMinterval vs REMpost.

The paper discussed mostly the second alternative, as stated in line 653: "REM propensity and its impact on the REM episode duration i s more closely associated with the time spent i n NREM sleep than with the combined time i n NREM sleep and wake." Authors limited the analysis of REMpre vs. inter-REMinterval to the results presented in Fig 1B. The problem of direct REMs episode duration with following amount of wakefulness (in inter-REM-interval) is the interference (masking) of feeding, drinking, exploring, etc, activities that obviously are outside sleep modulatory proceses, introducing loud noise in the timing of wakefulness with respect to the ultradian temporal frame.

I would suggest to explore the relationship of REMpre and REMpost with following interval duration and W duration by setting CDF constant.

The reviewer suggests to further explore the relationship between REM sleep, the REM propensity (quantified by the model CDF) and wakefulness during inter-REM (|W|). As mentioned by the reviewer, we already show that the correlation between the CDF at REM onset and the duration of the REM episode (REM_{post}) is not affected by |W| (**Fig 6C, S12 Figure**

D). With this analysis we studied the effect of wake on the REM propensity and its effects on REM_{post} (i.e. we studied the directionality |W| → CDF → REM_{post}). In a complementary analysis shown in **Reviewer Figure 3.1**, we test whether |W| is correlated with REM_{post} given a fixed CDF value at the onset of REM_{post}. For the analysis, we split the CDF values into 5 different ranges. In each case, we found either the p-value to be large or R^2 value to be small. Furthermore, there was no consistent relationship between |W| and REM_{post} for the different CDF ranges. This analysis further supports that |W| is not systematically related with REM_{post}, irrespective of the CDF at REM sleep onset.

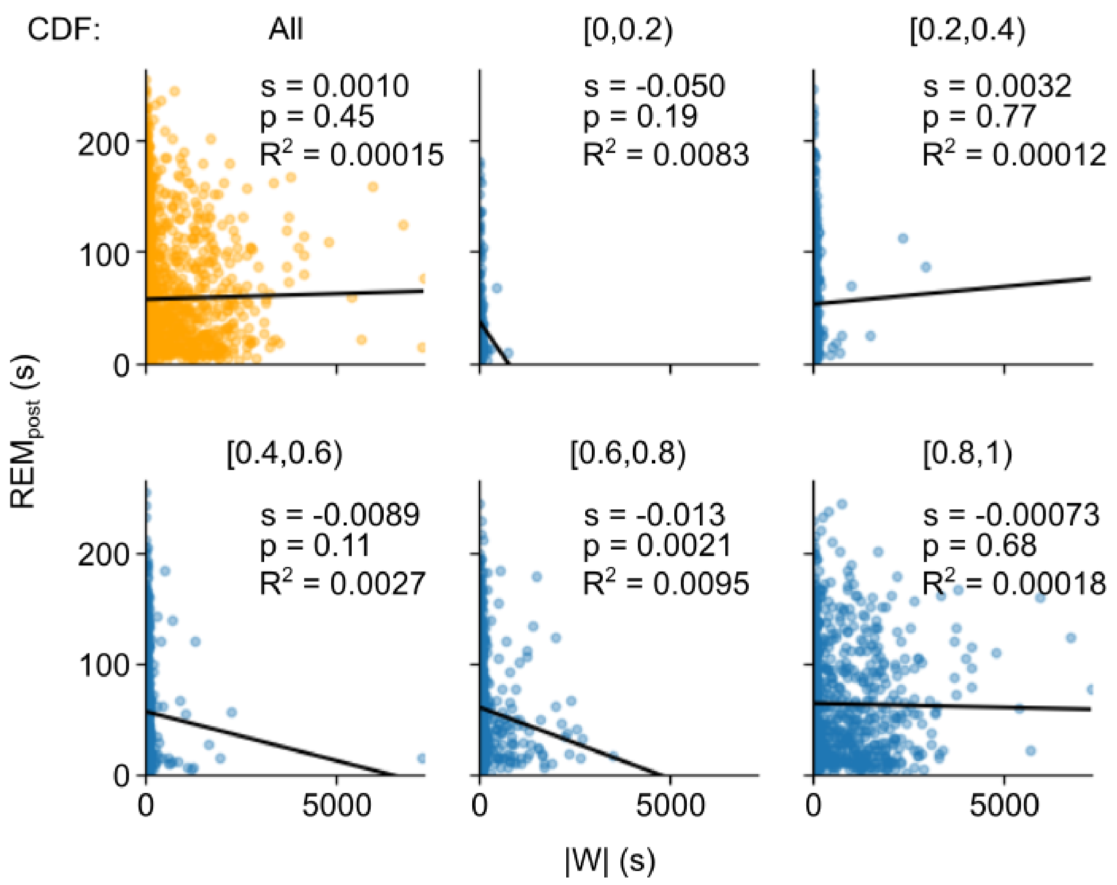

Reviewer Figure 3.1. Scatter plot of |W| vs. REM_{post} for varying values of the CDF at REM onset.

As suggested by the reviewer, we have also explored in more detail how the REM propensity (which depends on REM_{pre}) affects wake episodes (i.e. CDF → wake). Analogous to the analysis in **Fig 6C**, we calculated whether the CDF at the onset of a wake episode (instead of at the onset of a REM episode) correlates with the duration of this wake episode. The correlation

between the CDF at wake onset and the wake episode duration is not significant (**Reviewer Figure 3.2**), indicating that the current REM propensity does not influence the subsequent wake duration.

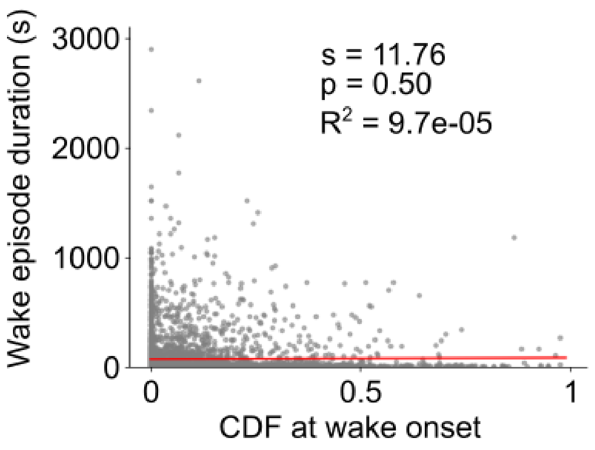

Reviewer Figure 3.2. Scatter plot of the CDF value at wake onset vs. wake episode duration.

2) Detailed information should be presented regarding the inter-individual variability. On the other hand, no details are presented with respect to the validation of automated sleep scoring (line 709: "we manually verified the automatic classification using a graphical user i nterface, visualizing the raw EEG, EMG signals, spectrograms and hypnogram").

To provide more details on inter-individual variability, we added a supplementary table (**S1 Table**) reporting the mean and standard deviation (across animals) for key parameters including the mean duration of REM sleep episodes, total duration of NREM (|N|), and Wake (|W|) during sleep cycles, the mean values of REM_{pre} for single and sequential cycles and the R^2-values for the correlation between REM_{pre} and |N|, |W|, or inter-REM. All these parameters are reported for both the light and dark phase.

At an initial stage of the project, we used the automatic scoring algorithm to explore this large data set. The same algorithm (programmed in Matlab) has been used in previous studies including (Chung et al., 2017; Weber et al., 2015). Based on our preliminary findings using the automatic scoring, we conceived the actual questions addressed in this study. As the exact detection of each single REM sleep period was essential for our analyses, we manually rescored the annotation to correct for errors, by visiting each single 2.5 s epoch in the hypnograms (a process which took months). We realized that the word "verify" (in "we manually verified the automatic classification using a graphical user interface") may imply a rather superficial inspection of the automatic classification. We therefore more accurately rephrased

the sentence as “we manually rescored the automatic classification using a graphical user interface to correct for errors” (page 27, line 781).

3) Results regarding night-day comparisons were not included in Discussion section? On the other hand I consider inappropriate the term “circadian” when referring to night-day comparisons. Circadian processes are estimated in circadian time or under specific experimental conditions that exclude local or zeitgeber time as main determinant, and is not the case in this experiment.

We agree that our use of the term “circadian” was imprecise. We therefore replaced “circadian” more appropriately with light and dark phase:

- We replaced the headline “Circadian modulation of REM sleep regulation” with “Changes in REM sleep regulation between light and dark phase” (page 19, line 537).
- We made the same change for the heading of **Fig 7**.
- Finally we replaced “to test for circadian differences in the regulation of REM sleep“ with “to test for differences in the regulation of REM sleep between the light and dark phase” (page 19, lines 539 - 540).

Using our model-based approach, we found two major differences in the regulation of REM sleep during light and dark phase: A delayed accumulation of REM propensity and a reduced frequency of sequential sleep cycles (**Fig 7**). As for the first finding, we proposed in the Discussion a potential mechanism, i.e. increased levels of orexin/hypocretin within the vlPAG during the dark phase may lead to an increased excitability of REM-off neurons (page 23, lines 660 - 666). In addition, we speculate now in the revised Discussion that increased levels of serotonin during the dark phase may underlie the reduced occurrence of sequential cycles (page 21, lines 616 - 620).

4) Presented results (particularly those related to sequential vs. single REMs) should be discussed in the light of evidences obtained in rats. In this sense I would suggest authors to review the following reference (Amici, et al. https://doi.org/10.1142/9781860947186_0008, in Parmeggiani &Velluti Eds. The Physiologic Nature of Sleep) where rat neurophysiological and vegetative aspects of REM sleep physiology are summarized. In this sense, there are interesting similarities between mice and rats in the neurophysiological description of sequential episodes.

We agree with the reviewer that our findings on the sequential REM sleep episodes in mice should be more thoroughly compared with previous results in rats. We have therefore added a new paragraph to the Discussion (page 21, lines 606 - 628) where we elaborate on previous findings about sequential sleep cycles in other species and compare findings in mice and rats. We agree with the reviewer that sequential cycles share many similarities in both species, including shorter average durations of the preceding REM episodes, reduced δ and σ power during NREM sleep, and reduced frequency of sequential REM periods during the dark phase (under normal laboratory temperature). These findings support the idea that sequential REM sleep episodes are regulated by the same mechanisms in both mice and rats.

Minor:

Place references 31 (Amici 1994) before reference 31 (Zamboni et al. 1999) , as the original description correspond of sequential/single episodes correspond to Amici.

As suggested by the reviewer, we now cite Amici et al. 1994 before Zamboni et al. 1999.

References

Chung, S., Weber, F., Zhong, P., Tan, C.L., Nguyen, T., Beier, K.T., Hörmann, N., Chang, W.-C., Zhang, Z., Do, J.P., et al. (2017). Identification of Preoptic Sleep Neurons Using Retrograde Labeling and Gene Profiling. Nature *545*, 477–481.

Weber, F., Chung, S., Beier, K.T., Xu, M., Luo, L., and Dan, Y. (2015). Control of REM sleep by ventral medulla GABAergic neurons. Nature *526*, 435–438.

Reviewer #4 (*reviewer's comments in italic*):

Summary: This is a very interesting paper that uses a wide range of statistical approaches to provide a highly detailed analysis of REM sleep in mice. As the authors suggest, their analyses establish a useful framework for characterizing "the ultradian regulation of REM sleep in health and disease." In addition, these analyses identify three major factors shaping ultradian regulation of sleep that have implications for the physiological basis of REM sleep regulation. My specific questions and concerns are listed below.

We thank the reviewer for the thorough review of our manuscript and providing clear and insightful feedback. The code used for our analyses is available on github (https://github.com/parksu92/REM_GMM) and we also deposited it on the data server (https://upenn.box.com/s/3zcesr4a7l7hgb9andmq4di4t6zvaoql) together with the sleep recordings.

Major concerns:

1. p.4, line 94: more detail/explanation is needed to support the claim that REM sleep sequences have been observed in multiple species including humans. The timescales of these sequences (and ultradian cycles in general) vary widely across species, so these terms should be defined carefully.

We agree with the reviewer that the concept of sequential REM sleep periods requires more explanation. We therefore added to the Discussion (page 21, lines 606 - 639) a new paragraph on sequential REM sleep episodes that more thoroughly explains how they were defined in other species, how their duration differs between these species and that compares our findings with those in rats. In addition, we mention now in the introduction (page 5, lines 94 - 97) that the distribution of the duration of inter-REM intervals is bimodal in rats, a finding which served as the basis to originally postulate the existence of two types of sleep cycles.

2. P. 25: the authors describe a semi-automatic scoring approach for determining sleep/wake behavior. Has this scoring approach been published previously? Validated against other rodent scoring?

At an initial stage of the project we used the automatic scoring algorithm to explore major trends in this large data set. The same algorithm (programmed in Matlab) has been used in previous studies including (Chung et al., 2017; Weber et al., 2015). Based on these preliminary findings, we formulated the main questions addressed in the present study. As the exact detection of each single REM sleep period was essential for our analyses, we manually rescored and, if necessary, corrected the annotation, by visiting each single 2.5 s epoch in the hypnograms (a process which took months). We realized that the word "verify" (in "we manually verified the automatic classification using a graphical user interface") may imply a more superficial inspection of the automatic classification. We therefore more accurately rephrased the sentence

as “we manually rescored the automatic classification using a graphical user interface to correct for errors” (page 27, line 781).

3. Related to the question about scoring, how was the 20 sec threshold for microarousals determined? This choice significantly affects the analysis through the definition of |N| and |W|.

Our threshold of 20 s for microarousals (MAs) lies in the range of thresholds used in other rodent studies. For example, (Pellegrini et al., 2016) used 16 s, (Léna et al., 2004) required that the duration of a MA lies between 5 s and 15 s, and (Watson et al., 2016) applied a 40 s threshold.
To further demonstrate that our key findings hold true, irrespective of the actual MA threshold, we fitted the model to our data set using different thresholds for MAs (30 s, 10 s, and 0 s, i.e. no MAs).
Based on the Lilliefors-corrected KS-test, we found that there is not enough evidence to reject the hypothesis that the data is drawn from a Gaussian mixture distribution for any of the tested MA thresholds (see new **S3 Table**). We then reperformed the analyses in **Figs 4** and **5** that directly depend on the chosen threshold and present the result in two additional supporting figures (**S8 Figure and S10 Figure**). Importantly, the key findings hold qualitatively true, irrespective of the applied MA threshold.

4. The authors should be careful with the language used in the interpretation of the model, particularly in the last paragraph of the Results on P. 18 and 19. Since the model has been fit to the data, the model parameters describe the data but do not explain features such as an increased percentage of single cycles or contribute to an increase in NREM relative to REM sleep. Similarly, on p. 2, line 24: it seems too strong to say that the model “confirms” the existence of two types of sleep cycles; it would be better to say “supports” here.

We agree with the reviewer that in some instances our language was too imprecise when interpreting the model. We therefore made the following changes in the text:

- In the last paragraph of the Results, we replaced “explaining the increased percentage of single cycles” with “reflecting the increased percentage of single cycles” (page 19, line 571).
- Similarly, we replaced “As single cycles contain less REM sleep relative to NREM sleep, the increase of k_{long} during the dark phase contributes to the decrease in REM sleep.” with “As single cycles contain less REM sleep relative to NREM sleep, their reduced frequency during the dark phase, mirrored in the increase of k_{long}, contributes to the decrease in REM sleep.” (page 19, lines 572 - 575).
- We replaced the sentence “Second, the mean of the long Gaussian distribution, μ_{long}, was larger in the dark phase, also contributing to the increase in NREM relative to REM sleep (**Fig 7D,E**).” with “Second, the mean of the long Gaussian distribution, μ_{long}, was larger in the dark phase, indicating a delayed increase in the likelihood of NREM to REM sleep transitions, a second factor contributing to the increase in NREM relative to REM sleep (**Fig 7D,E**).” (page 20, lines 575 - 577).

- As suggested by the reviewer, we replaced ‘confirms’ with ‘supports’ in the Abstract (page 2, line 24).
- In the Discussion, we replaced ‘further confirming that REM sleep’ with ‘further supporting the notion that REM sleep’ (page 24, line 649)

Minor concerns:

1. Some of the citations in the Introduction seem a little sparse; e.g.,
a. P.3, line 67: citations to REM networks should include work from Yang Dan’s group such as Chen et al., Neuron, 2018
b. P. 4, line 79: citations to REM homeostat should include Franken et al. (ref [51])

We included (Chen et al., 2018) as an example for a study describing hypothalamic REM sleep control circuits and now cite (Franken, 2002) when introducing the idea of the hourglass-type ultradian process for REM sleep regulation; we apologize for this oversight.

2. P. 6, line 141: more detail is needed for the construction of the probability densities in Figure 2B; I am interpreting the terminology “REM_pre splits” to refer to subsets of REM_pre, but I am unclear on the significance of these subsets. How were the 30 second bins chosen? Were there similar numbers of REM_pre episodes in each subset? Approximately how many?

Yes, we divided REM sleep episodes based on their duration (REM_{pre}) into 8 consecutive equal-width intervals (or bins) of duration 30 s. For each interval, we then used all REM periods falling into this interval to compute the histogram and probability density over ln(|N|). We noticed that the terminology REM_{pre} split may not be clear enough and instead of ‘splits’, we now use the term ‘bin’. In addition, we have modified the text in the Results (page 6, lines 144 - 148) and added more explanation to the Methods (page 28, lines 790 - 795).

Our rationale for why we chose 30 s intervals is that this bin size is small enough to resolve the dependence of the conditional distributions on REM_{pre}, but large enough to contain a sufficient number of data points (even for large REM_{pre} values) to reliably estimate the probability densities. As the distribution of REM sleep durations has a long tail, the number of episodes falling into each subset decreases as REM_{pre} increases. The exact numbers of REM periods falling into each interval are listed in **S2 Table**.

3. P. 8, line 198-201: the interpretation of the duration of the refractory period as a function of REM_pre needs clarification. Does this correspond to the increased black region below the red band in the heat map in Fig 2E?

The formal definition of the refractory period is provided later in the manuscript in **Fig 4A, B** (The black line in **Fig 4A** divides single sleep cycles into their refractory and permissive period). We believe that the heatmap in **Fig 2E** provides an intuitive summary of the key concepts of our study (single vs. sequential cycles; refractory vs. permissive period) that are then analyzed in the remainder of the paper. Therefore while discussing the heatmap, we already introduce these

concepts. But we understand that the reader, not knowing what will be discussed later on, may expect more explanations at this point. To address this issue, we first dropped two observations that are difficult to recognize in the heatmap and that are discussed in depth in other sections of the paper: The standard deviation of the duration of single cycles “decreases with increasing REM_{pre}“ and the duration of the refractory periods “lasts roughly twice the duration of REM_{pre}“. Second, we added an explicit reference to **Fig 4**, when introducing the refractory period to make clear that more details are provided later in the manuscript (page 8, lines 206 - 209).

4. P. 9, lines 216 – 223: this comparison with previous work may be better in the Discussion.

Given that this paragraph compares our findings with another study, we agree that this section would also fit well into the Discussion. On the other hand, the arguments in this paragraph are quite technical in nature, including a reference to **S6 Figure**; that is why we felt that it fits slightly better into the Results section.

5. P. 9, line 229: what is the |N| duration associated with optimal separation in Figure 3A (right)? How does this threshold vary with |N|, and using this threshold, what percentage of sequential cycles are misclassified as long and vice versa (for different values of |N|)?

As shown in **Fig 3A** left, the threshold of |N| that separates sequential from single sleep cycles (black line) varies with REM_{pre} (and not with |N|). **Fig 3A** right illustrates as an example how this threshold (optimal separation between short and long distribution) was determined for REM_{pre} = 30 s.
As depicted in **Reviewer Figure 4.1A**, the intersection of the distributions for short and long cycles corresponds to the optimal threshold separating sequential form single cycles (note that the two distributions are weighted by the factors (1-k_{long}) and k_{long}, respectively). The value **B/(A+B)** corresponds to the likelihood that a sequential cycle might be classified as a single cycle. The resulting misclassification rate is depicted in **Reviewer Figure 4.1B** as a function of REM_{pre}.

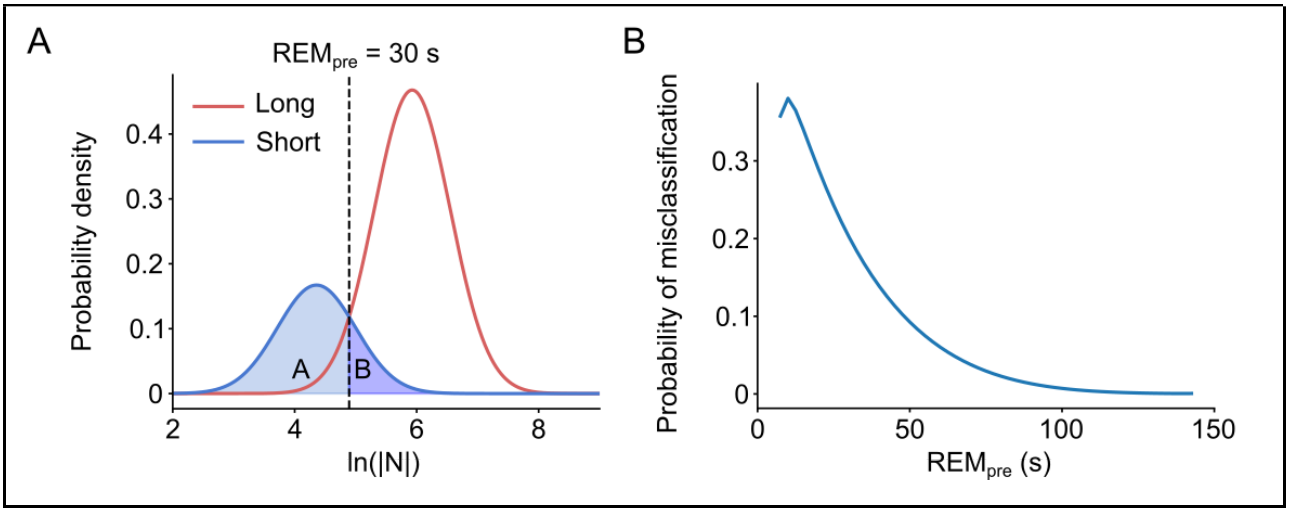

Reviewer Figure 4.1 Probability of misclassifying cycles.
(A) Illustration of how the threshold separating sequential from single cycles was determined for REM_{pre} = 30 s. The value B/(A+B) corresponds to the probability of misclassifying a sequential cycle as a single cycle.
(B) The probability of misclassifying a sequential cycle as a single cycle (B/(A+B)) as a function of REM_{pre}.

6. P. 9, line 235: the interpretation of a sequence of REM sleep with only one cycle as comprising 2 REM periods is given in the caption of Figure 3 caption but not in the text. It would be useful to define in both places.

We now also define one cycle as comprising two REM periods in the text (page 9, lines 242 - 244).

7. P. 17, line 498: the authors claim that the REM duration is more closely correlated with the CDF than the preceding NREM duration. Is this conclusion supported by a formal analysis of the correlations (e.g., Williams correlation test)?

As suggested by the reviewer, we performed Williams' correlation test. The test formally confirmed that the correlation between the CDF and REM_{post} is larger than the correlation between |N| and REM_{post} ($t = 17.5865$, $p = 0.0$). We now cite the test results in the text (page 17, lines 503 - 506).

8. P 18, line 508: careful language should be used when talking about circadian differences; although circadian modulation of REM sleep has been established in other work, these results focus on light/dark differences.

We replaced the term "circadian" with the more correct wording "light and dark" throughout the manuscript.

9. P. 18, line 537-538: it would help to remind the readers that k_long is the weighting parameter for the long REM bouts in the Gaussian mixture model.

For better readability, we again mention the definition of k_{long} in the text (page 19, line 570).

10. P. 20, line 583: the sleep phase N1 is increased in narcolepsy, and, although this light phase of sleep is associated with transitions to wake, fragmentation of N1 is not increased in narcolepsy (type 1) compared to controls (Maski et al., SLEEP, 2021).

We thank the reviewer for this clarification and pointing us to this recent publication. We dropped the part of the sentence stating that N1 is "particularly susceptible to sleep fragmentation in sleep disorders such as narcolepsy" (page 22, lines 651 - 652).

References

Chen, K.-S., Xu, M., Zhang, Z., Chang, W.-C., Gaj, T., Schaffer, D.V., and Dan, Y. (2018). A Hypothalamic Switch for REM and Non-REM Sleep. Neuron *97*, 1168-1176.e4.

Chung, S., Weber, F., Zhong, P., Tan, C.L., Nguyen, T., Beier, K.T., Hörmann, N., Chang, W.-C., Zhang, Z., Do, J.P., et al. (2017). Identification of Preoptic Sleep Neurons Using Retrograde Labeling and Gene Profiling. Nature *545*, 477–481.

Franken, P. (2002). Long-term vs. short-term processes regulating REM sleep. J. Sleep Res. *11*, 17–28.

Léna, C., Popa, D., Grailhe, R., Escourrou, P., Changeux, J.-P., and Adrien, J. (2004). β2-Containing Nicotinic Receptors Contribute to the Organization of Sleep and Regulate Putative Micro-Arousals in Mice. J. Neurosci. *24*, 5711–5718.

Pellegrini, C., Lecci, S., Lüthi, A., and Astori, S. (2016). Suppression of Sleep Spindle Rhythmogenesis in Mice with Deletion of CaV3.2 and CaV3.3 T-type Ca2+ Channels. Sleep *39*, 875–885.

Watson, B.O., Levenstein, D., Greene, J.P., Gelinas, J.N., and Buzsáki, G. (2016). Network Homeostasis and State Dynamics of Neocortical Sleep. Neuron *90*, 839–852.

Weber, F., Chung, S., Beier, K.T., Xu, M., Luo, L., and Dan, Y. (2015). Control of REM sleep by ventral medulla GABAergic neurons. Nature *526*, 435–438.
