# Supplementary material for: A probabilistic model for the ultradian timing of REM sleep in mice
Source: PLoS Comput Biol. 2021 Aug 25;17(8):e1009316. doi: 10.1371/journal.pcbi.1009316 (PMC8423363; doi:10.1371/journal.pcbi.1009316)
Supplement: Attachment — Submitted filename: response_to_reviewers.pdf [file pcbi.1009316.s018.pdf]

Reviewer #1: Thank you for carefully addressing all my comments. This is an excellent paper and a significant contribution to our understanding of REM/NREM sleep cycles.

We again thank the reviewer for the encouraging comments and insightful suggestions to improve our manuscript!

Reviewer #2: Thanks for the interesting discussion. I re-iterate that I am not scoring your manuscript even though I think your data are great. I just don't have the expertise to evaluate your modeling that is a major portion of the paper. However, I was trying to convince you that you have a perfectly good physiological explanation of your data without invoking a mysterious refractory phase, which the data do not support. That explanation is classical homeostatic regulation, which your data do support. The definition of refractory is to be insensitive to a stimulus that was previously effective. A neuron is refractory to depolarizing input immediately following an action potential. A man is refractory to sexual stimulation following orgasm. If you went out into the cold without a coat and started to shiver, but then put on a coat and stopped shivering, are you refractory to cold? No, you have just eliminated the stimulus. At one point you also seem to accept someone else's description of the REM/NREM process as being like an hour glass --- no it is not. Maybe an old fashioned ice box is like an hour glass as the ice melts, but a modern refrigerator is a regulated system. The NREM REM cycles are regulated homeostatically as shown by both our results. If you are looking for a function of REM, you are more likely to find it by looking for the feedback information relating NREM to REM, and not in a supposed mechanism of refractoriness.

As we showed, the brief REM episodes are likely to occur at any time in the classical sleep cycle, but are increasingly likely as the cycle continues. Also, a brief REM episode is likely to be followed soon by another one, and so on with increasing rapidity. These brief REM episodes are failures to maintain REM, and when they fail they do not dissipate much REM propensity, so they come again sooner and sooner until a REM bout is sustained and dissipates the REM need.

Physiologically, we need to be looking for what process is exhausted or built up during the hyperpolarized NREM state that has to be reversed during REM. That is investigating a homeostatic process and not a refractory mechanism.

You can interpret your data anyway you want in your paper, of course. But, I just want you to try thinking of your beautiful data in a physiological way.

Thank you again for your comments and for clarifying your concerns with our interpretation of the data.

Our definition of the “refractory” period is derived purely statistically based on data of spontaneous sleep in mice. Therefore, our analysis does not directly speak to the physiological mechanisms underlying this interval of NREM sleep. But, out of consistency with previous studies using the term “refractory” to describe an interval of NREM sleep following REM sleep during which transitions to REM sleep are unlikely (Borbély, 1980; Franken, 2002; Le Bon, 2020, 2021; Ocampo-Garcés et al., 2020) we decided to also use it for our study.

Although our use of the term may be too lenient, we do provide a strict statistical definition (Fig 4A,B). Moreover, in the revised Discussion, we now stress that the refractory period is derived from statistical analysis and mention that further experiments are needed to test whether it is really “refractory” in the sense that REM sleep-promoting stimuli cannot trigger REM sleep (page 21, lines 625 - 632).

References

Borbély, A.A. (1980). Effects of Light and Circadian Rhythm on the Occurrence of REM Sleep in the Rat. Sleep *2*, 289–298.

Franken, P. (2002). Long-term vs. short-term processes regulating REM sleep. J. Sleep Res. *11*, 17–28.

Le Bon, O. (2020). Relationships between REM and NREM in the NREM-REM sleep cycle: a review on competing concepts. Sleep Med. *70*, 6–16.

Le Bon, O. (2021). An Asymmetrical Hypothesis for the NREM-REM Sleep Alternation—What Is the NREM-REM Cycle? Front. Neurosci. *15*.

Ocampo-Garcés, A., Bassi, A., Brunetti, E., Estrada, J., and Vivaldi, E.A. (2020). REM sleep-dependent short-term and long-term hourglass processes in the ultradian organization and recovery of REM sleep in the rat. Sleep *43*, 1–13.

Reviewer #3: I have no further concerns.

We want to thank the reviewer again for the thorough assessment of our study and insightful comments.

Reviewer #4: Summary: This is a very interesting paper that uses a wide range of statistical approaches to provide a highly detailed analysis of REM sleep and the impact of REM sleep on NREM sleep in mice. The authors have addressed my previous comments in the revised manuscript, and the new Discussion subsection on Sequential Sleep Cycles is a great addition. One few minor suggestion:

Lines 612-615: The authors state that "In rats and, as shown here, in mice the average duration of REM sleep episodes preceding a sequential cycle is on average shorter than that before a single cycle, and the frequency of sequential cycles is reduced during the dark period [33]." Is this comment actually restricted to the REM sleep episodes preceding these events or should it describe REM sleep episodes within sequential cycles compared to REM sleep episodes in a single cycle? If the authors are highlighting a feature of the first REM sleep episode in a sequential cycle, it would be helpful to clarify this point.

We thank the reviewer for pointing out a potential misunderstanding, which indeed requires clarification. We have therefore adjusted the mentioned sentence "In rats and, as shown here, in mice the average duration of REM sleep episodes preceding a sequential cycle is on average shorter than that before a single cycle…" to "In rats and, as shown here, in mice the durations of REM sleep episodes at the beginning of sequential cycles are on average shorter than those at the start of single cycles…" (Page 20, lines 583 - 585).
