# Supplementary material for: A probabilistic model for the ultradian timing of REM sleep in mice
Source: PLoS Comput Biol. 2021 Aug 25;17(8):e1009316. doi: 10.1371/journal.pcbi.1009316 (PMC8423363; doi:10.1371/journal.pcbi.1009316)
Supplement: Attachment — Submitted filename: Review of REM-NREM paper.docx [file pcbi.1009316.s016.docx]

Review of “A probabilistic model for the ultradian timing of REM sleep in mice”by S-H Park et al.

I am really pleased to see this strong support for the ideas we advanced many years ago – your reference 19. I cannot offer any expertise in the review of your mathematical modeling, but I can raise questions and suggestions that you might find useful to consider.

First, there is an important difference between your basic approach to the EEG analysis and ours, and this difference could be at the root of some of our apparent conflicts in interpretation. Please check our methods paper, Benington, Kodali, Heller (1994) Scoring transitions to REM sleep in rats based on the EEG phenomena of Pre-REM sleep: an improved analysis of sleep structure. Sleep 17(1):28-36. We both used spectral analysis of key frequency bands, but the important difference is that we used those signatures to identify transitions to REM even when no scorable REM ensued. I believe you used a scoring threshold of 30 sec of scorable REM, but maybe I am wrong. These NRTs, as we called them, are an excellent indicator of REM pressure.

Here are some comments based on figures in your Ref 19. First, if you compare the distribution of NREM episode durations using only REM episodes > than 30 sec, vs. looking at the durations of NREM episodes as a function of all inter NRT intervals, you get very different distributions. I think that the latter analysis yields a more likely representation of a homeostatic system.

Second, if you use the NRT scoring for the brief REM sleep episodes, you see a beautiful relationship indicating the build up of REM pressure.

Third, you postulate the existence of a “refractory period” with no evidence for such a phenomenon other than the data based on your rules of analysis. If you use NRTs rather than sustained REM episodes, there is no indication of a refractory period. Consider the following data also from ref. 19. The probability of a brief REM episode as determined by an NRT is the same no matter whether the sleep cycle has just begun (ie, no refractory period), is midway, or is nearing completion.

I know this is an old paper and does not make use of sophisticated modeling, but I think the data are reflecting the true nature of REM – NREM interactions more precisely than the analysis you have used that has led you into proposing a refractory period for which there is no evidence.

Comments specific to text.

Starting on line 80. A lot rides on this introduction of the concept of refractory period which you acknowledge is not defined, nor is there any evidence. The results cited fit perfectly well with a homeostatic model – a long REM episode more fully discharges the need for REM and is therefore permissive of NREM.

Line 104. All REM-NREM cycles fit on the same relationship, so why divide them into two groups?

Lines 211-215. I disagree.

Lines 274-276. This is to be expected because at the transition to NREM, it takes about a minute to reach full delta power. If unsuccessful attempts to enter REM occur frequently, the consequence will be lower delta power. Without knowing what the trigger for REM sleep transition is, it is not possible to experimentally test the concept of a refractory period. However, you show nicely that the REM-NREM relationship is homeostatic, and the data can be interpreted more parsimoniously by that homeostatic relationship. The problem is that you have set an arbitrary definition of a REM episode rather than recognize the existence of a transition to REM in the absence of that arbitrary value.

You have great data, but because I do not feel qualified to evaluate your modeling, and because you might consider my major criticism just a defense of another view point, I am submitting the review without a recommendation, but I hope you will take my comments into consideration.

Best,

Craig Heller
